# Pantothenate regulates feeding and reproduction in the malaria vector *Anopheles stephensi*, with patterns dependent on supplementation scheme and parental nutrition

**DOI:** 10.1186/s13071-025-06959-w

**Published:** 2025-08-04

**Authors:** Megan E. Dobson, Hannah L. Kaylor, Sydney L. Pruett, Jessica Brady, Kayla Savoie-Penton, Jun Isoe, Yared Debebe, Michael A. Riehle, Shirley Luckhart

**Affiliations:** 1 Department of Biological Sciences, University of Idaho, Moscow, ID 83843 USA; 2 Department of Entomology, Plant Pathology, and Nematology, University of Idaho, Moscow, ID 83843 USA; 3 Department of Entomology, University of Arizona, Tucson, AZ 85721 USA

**Keywords:** Anopheles stephensi, Pantothenate, Vitamin B5, Reproduction, Malaria, Fecundity, CoA

## Abstract

**Background:**

Pantothenate (Pan), or vitamin B5, is the substrate for biosynthesis of coenzyme A (CoA), an essential cellular cofactor involved in many metabolic processes. Our previous studies demonstrated that Pan availability influences a broad range of traits across multiple species, including malaria parasite development in the mosquito *Anopheles stephensi*. Accordingly, restricting Pan availability during parasite development may be a viable strategy for malaria control. However, the physiological roles of Pan in *A. stephensi* remain unclear. In these studies, we investigated the effects of Pan supplementation on this globally important malaria vector.

**Methods:**

Female *A. stephensi* were supplemented with Pan via either water, which, similar to plant nectar, is directed to the crop and then slowly released into the midgut, or blood, which transits directly to the midgut for digestion. The effects of provisioning on subsequent blood feeding behavior, reproduction, and offspring sex ratio were assessed. We evaluated these traits across multiple generations, with and without additional supplementation of offspring.

**Results:**

Our findings revealed that Pan regulates vectorially important traits in concentration-, delivery-, and age-dependent ways. The greatest effects of Pan provisioning were on reproduction. The unsupplemented offspring of mothers supplemented with Pan via water exhibited increased fecundity, indicating transgenerational effects from supplemented mothers. However, when Pan was provisioned in blood, only mothers and their supplemented offspring exhibited altered reproduction.

**Conclusions:**

Our work establishes the importance of Pan in *A. stephensi* reproduction and provides a foundation for investigating the transgenerational effects of Pan and CoA on mosquito physiology. These observations suggest that targeting Pan-CoA biology in *Anopheles* spp. could provide opportunities for novel mosquito control strategies.

**Graphical Abstract:**

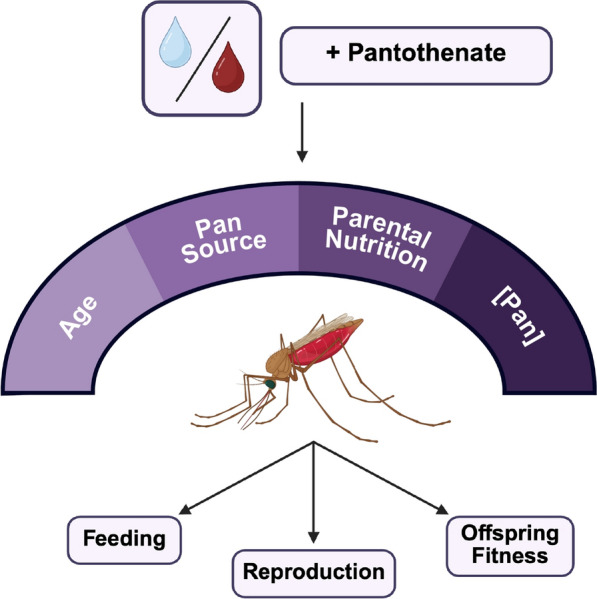

**Supplementary Information:**

The online version contains supplementary material available at 10.1186/s13071-025-06959-w.

## Background

Despite significant progress, malaria remains a major global health challenge. Since 2020, worldwide cases have increased annually, reaching an estimated 263 million cases in 2023 [1]. This disease, caused by infection with *Plasmodium* parasites transmitted by female *Anopheles* mosquitoes, is becoming increasingly difficult to control. Insecticide-resistant mosquitoes, drug-resistant parasites, and the rapid spread of the highly competent vector *A. stephensi* into Africa are threatening existing interventions [2–4]. Accordingly, we must develop novel strategies for malaria control, including new mosquito-targeted interventions.

*Plasmodium* spp. depend on nutrients from both human and mosquito hosts to develop and propagate. One transmission-blocking strategy is starving the parasite of these essential nutrients inside the vector. The coenzyme A (CoA) biosynthesis pathway is a promising target for this intervention strategy [5–7]. The ubiquitous cellular cofactor CoA is synthesized from pantothenate (Pan or vitamin B5) via conserved pathways in the human host, mosquito vector, and malaria parasite [8, 9]. CoA is involved in critical enzymatic processes, including fatty acid biosynthesis, protein acetylation, and cellular respiration [10]. Malaria parasites cannot synthesize Pan de novo, and mosquito-stage parasites cannot acquire preformed CoA from the insect host; therefore, they must obtain Pan from the mosquito to survive and develop into infectious sporozoites [6, 11, 12].

CoA biosynthesis begins with the rate-limiting enzyme pantothenate kinase (PanK), which catalyzes the phosphorylation of Pan [9]. Our previous studies demonstrated that provisioning of young (3–5-day-old) female *A. stephensi* with the PanK activating pantazine drug PZ-2891 significantly reduced Pan availability in adult mosquitoes, thereby reducing infection success of diverse malaria parasite species in *A. stephensi* [6, 13]. Subsequent studies determined that provisioning of PZ-2891 decreased whole-body Pan but did not affect reproduction in 3–5-day-old *A. stephensi* [13]. We showed that these young mosquitoes possessed high levels of endogenous Pan [14]. However, in the absence of any dietary sources, Pan levels in adult females decreased with age, reaching the lowest level at 14 days post-eclosion [14]. These data suggested that gut microbiota were not a primary source of Pan for adult mosquitoes [14].

Pan is available to adult mosquitoes through nectar—a common source of this vitamin across diverse plant species [R. Vannette and C. Rering, unpublished data, 15]—which is consumed by both male and female mosquitoes. Nectar is a chemically complex and variable resource, with its composition influenced by plant species, environment, and associated microbial communities [15, 16]. In addition to carbohydrates, nectar contains amino acids, organic acids, and micronutrients that play roles in mosquito reproduction, behavior, and survival [17, 18]. However, the specific plants that serve as nectar sources for *A. stephensi* remain largely undescribed, as does the full extent of the biochemical diversity of the associated nectar meals.

Despite the low levels of Pan in human blood (0.00038 g/L to 0.0013 g/L in healthy adults [19], female *A. stephensi* acquire some Pan during blood feeding [14]. Pan is likely essential to mosquito reproduction. During egg development, Pan is allocated to the ovaries and transported intracellularly, processes associated with enhanced follicular development [14]. When Pan uptake was inhibited, follicular development was significantly reduced [14]. Intriguingly, provisioning supplemental Pan via an artificial blood meal to older, Pan-depleted females reversed the decline in fecundity associated with aging [14, 20]. Together, these findings suggest that Pan is an important and limiting nutrient in *A. stephensi* reproduction.

Across a broad range of species, alterations to PanK, Pan, and CoA levels impact several life history traits, including offspring sex ratio, feeding behavior, survival, and reproduction [21–26]. These traits are relevant to vectorial capacity and population dynamics across multiple mosquito species including those in the genus *Anopheles* [27, 28]. In *A. stephensi*, the provisioning of a small molecule PanK inhibitor to adult females decreased egg production [13]. Additionally, the nutritional environment of the parental or zero filial generation (F0) in multiple mosquito genera has been reported to impact the fitness and fecundity of offspring (F1) [29, 30]. These fitness differences may be partially attributed to variable nutrient provisioning from mother to offspring [29]. In the context of Pan and CoA, Yu et al. [31] reported that female *Drosophila melanogaster* provisioned CoA and its precursors to their developing offspring. These maternally provisioned resources were sufficient to allow for development to the second instar larval stage with no alternative sources of CoA [31].

CoA is used to synthesize acetyl-CoA, which is crucial for protein acetylation, a process that regulates many cellular functions. The abundance of CoA influences the amount of protein acetylation [32]. Notably, acetyl-CoA can freely pass through nuclear pores into the nucleus, where it functions as the substrate for lysine acetyltransferase (KAT)-dependent acetylation of histone proteins [10]. This process regulates gene expression, impacting cell activities from metabolism to proliferation [32]. With relevance to lifespan and reproduction, the silencing of several KAT genes in the pea aphid *Acyrthosiphon pisum* increased survival and the number of offspring produced by females [33], suggesting a variety of mechanisms whereby altered Pan, PanK, and CoA levels can have transgenerational effects.

Despite the physiological importance of Pan and CoA, the impacts of dietary Pan on adult female mosquitoes are largely unexplored [7]. In these studies, we investigated the effects of provisioning Pan to *A. stephensi* in water (alongside a sugar cube for nutrition) and in blood to adult female mosquitoes. We assessed the effects of Pan provisioning on fecundity, blood feeding behavior, and offspring sex ratio across multiple reproductive cycles, generations, and mosquito ages. Our findings demonstrate that the impacts of Pan provisioning vary with delivery method, concentration, parental supplementation status, and adult female age.

## Methods

### Mosquito rearing

*A. stephensi* Liston (Indian strain) used in these experiments were maintained at the University of Idaho and derived from eggs acquired in 1998 from the Department of Entomology at Walter Reed Army Institute of Research (WRAIR; Washington, D.C., USA). All mosquito life stages were maintained at 28 °C, 80% humidity, and under 12 h light–dark cycles (lights on at 08:00 h, lights off at 20:00 h). For colony maintenance, mosquitoes were fed on CD-1 mice (Envigo, St. Louis, MO, USA) sedated with ketamine (50 mg/kg) and xylazine (5 mg/kg). Mouse protocols performed at the University of Idaho were approved by the Institutional Animal Care and Use Committee in accordance with federal regulatory guidelines and standards (University of Idaho IACUC-2023-08 protocol, approved 27 March 2023). Unless otherwise stated, adult mosquitoes were maintained ad libitum on 10% sucrose-soaked cotton balls (replaced every other day) and housed in 2-L polypropylene containers topped with mesh screening. Eggs produced by experimental mosquitoes were washed into 5-L Nalgene pans with 500 mL of water. Larvae were provided with a solution of 2% Sera Micron fry food and active dry baker’s yeast in a 2:1 ratio for 3 days, after which they were fed Purina Game Fish Chow pellets until pupation.

### Pan supplementation via water

**Fig. 1:**
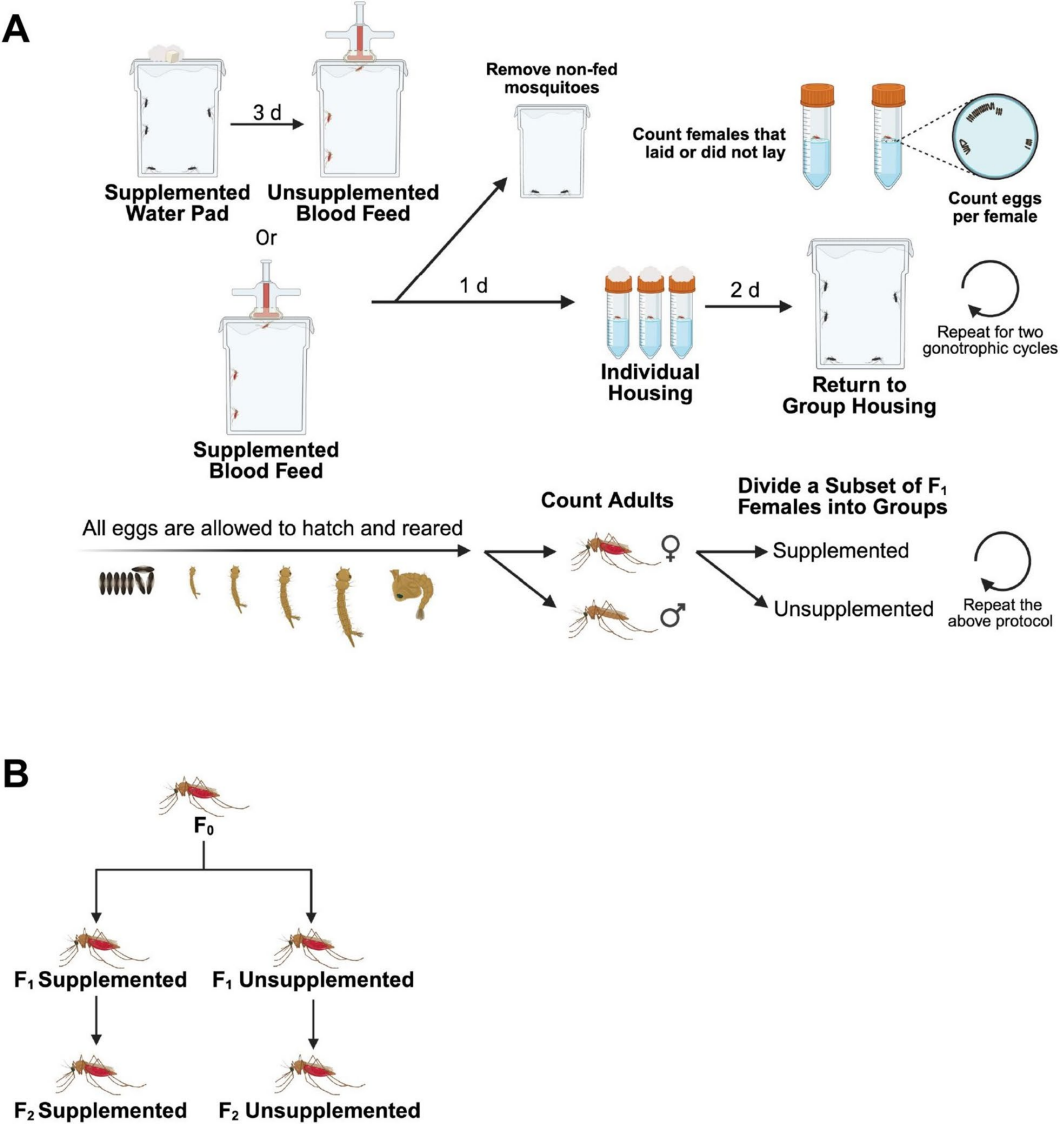
Experimental design for assessing the effects of Pan supplementation on female *A. stephensi* across multiple generations. (**A**) Adult females were provided with either Pan-supplemented water followed by unsupplemented blood or with Pan-supplemented blood. Following blood feeding, non-fed females were removed, and oviposition was monitored. Eggs per female were recorded, and larvae were reared to adulthood. Offspring were then divided into Pan-supplemented and unsupplemented groups (**B**), and the protocol (**A**) was repeated for subsequent generations

For several experiments, Pan was provided to mosquitoes via water-soaked cotton balls alongside a sugar cube prior to blood feeding (Fig. 1A). For control and treatment groups, 80–120 adult female mosquitoes (F0) were housed in 2-L polypropylene cartons topped with mesh screening. Offspring from these females (F1) and a subsequent generation (F2) were used in further studies. Cotton pads were changed twice daily, once in the morning between 08:00 and 10:00 h and once in the evening between 16:00 and 18:00 h. Control groups were provided with filtered water, and treatment groups received filtered water supplemented with 0.05, 0.5, 5, or 50 g/L d-pantothenic acid hemicalcium salt (Sigma-Aldrich, Saint Louis, MO, USA). These concentrations surpass what mosquitoes might encounter through nectar feeding, allowing us to evaluate the effects of highly elevated Pan levels. These dosages also encompass the broad range of Pan concentrations provisioned in studies with other invertebrates [21, 25]. Mosquitoes were allowed to complete two gonotrophic cycles (GC1 and GC2). Fresh Pan treatments were prepared 1 day prior to each provisioning period and stored at 4 °C. Mosquitoes were allowed access to supplemented water for 3 days prior to receiving an unsupplemented artificial blood meal. A subset of F1 females received the same Pan treatment as their mothers and were termed “supplemented.” Another subset of F1 females received no supplementation and were termed “unsupplemented” (Fig. 1B). Each biological replicate used a separate cohort of *A. stephensi*.

### Artificial blood meal delivery

All soaked cotton balls and sugar cubes were removed 45 min prior to blood feeding. Mosquitoes were offered a blood meal composed of a 1:1 vol/vol ratio of washed human red blood cells (RBCs) and heat-inactivated human serum (Grifols Bio Supplies, Inc., Los Angeles, CA, USA) for 30 min. Blood meals were offered via 5-mL glass membrane feeders with collagen membranes at 37 °C between 12:30 and 15:30 h for each experiment to maintain consistency.

### Pan supplementation via blood meal

Pan was provisioned to mosquitoes via artificial blood meal as described above (Fig. 1A). d-pantothenic acid hemicalcium salt was added to blood at a final concentration of 0.05, 0.1, 0.5, or 1 g/L, while controls were provided blood without Pan. A subset of F1 females received the same Pan treatment as their mothers and were termed “supplemented.” Another subset of F1 females received no supplementation and were termed “unsupplemented” (Fig. 1B). All mosquitoes were allowed to complete two gonotrophic cycles. Each biological replicate used a separate cohort of *A. stephensi*.

### Assessing effects of Pan supplementation on the tendency of *A. stephensi* to blood feed

Female mosquitoes were treated either via water supplementation before a blood meal or via blood meal as described above. Water-supplemented mosquitoes were offered an unsupplemented blood meal following the 3-day provisioning period. For the first blood feed, 80–120 female mosquitoes (5–7-days-old) housed in 2-L polypropylene containers (group housing) were offered an artificial blood meal. For the second blood meal, all remaining mosquitoes (13–15-days-old) were offered a blood meal. Following each blood meal, any nonfed females were removed and quantified. For Pan provisioning in water, we performed six biological replicates for F0 (GC1 = 2,181 females; GC2 = 1,689 females), five biological replicates for supplemented F1 (GC1 = 2,078 females; GC2 = 1,350 females), and four biological replicates for unsupplemented F1 (GC1 = 1,727 females; GC2 = 1,068 females). For Pan provisioning in blood to F0, we performed five biological replicates for GC1 (*N* = 2,547 females) and four biological replicates for GC2 (*N* = 1,337 females). We completed four biological replicates for supplemented F1 (GC1 = 1,992 females; GC2 = 1,132 females) and three biological replicates for unsupplemented F1 (GC1 = 1,462 females; GC2 = 856 females).

### Assessing effects of Pan supplementation on *A. stephensi* oviposition and clutch size

Up to 75 bloodfed females from each treatment group were moved to individual housing (50-mL conical tubes with 2.5-mL water in the bottom) at 24 h following blood feeding to allow for oviposition. After 48 h, females were transferred back to group housing. The numbers of females that laid eggs and the numbers of eggs in each tube were counted and recorded for each treatment group. Females that died in the tube without laying eggs were removed from further analyses, as we could not determine whether they died prior to having the opportunity to oviposit. Data from females that died in the tube and laid eggs, however, were included in further analyses. For Pan provisioning in water, we performed six biological replicates for F0 (GC1 = 1,763 females; GC2 = 1,107 females), five biological replicates for supplemented F1 (GC1 = 1,460 females; GC2 = 895 females), and four biological replicates for unsupplemented F1 (GC1 = 1,214 females; GC2 = 750 females). For Pan provisioning in blood to F0, we performed five biological replicates for GC1 (*N* = 1,640 females) and four biological replicates for GC2 (*N* = 979 females). We completed four biological replicates for supplemented F1 (GC1 = 1,325 females; GC2 = 839 females) and three biological replicates for unsupplemented F1 (GC1 = 994 females; GC2 = 568 females).

### Assessing effects of Pan supplementation on *A. stephensi* offspring sex ratio

Following oviposition, eggs produced by F0 and F1 females were washed into 5-L Nalgene pans with 500 mL of water. These offspring were reared under the same conditions as their parents. Once pupation began, pupae were collected every 24 h until fewer than 20 larvae remained. Following adult emergence, mosquitoes were quantified and sexed by visual observation. For Pan provisioning in water, we performed six biological replicates for F0 (GC1 = 32,227 offspring; GC2 = 17,179 offspring), four biological replicates for supplemented F1 (GC1 = 24,813 offspring; GC2 = 25,659 offspring), and three biological replicates for unsupplemented F1 (GC1 = 22,053 offspring; GC2 = 21,494 offspring). For Pan provisioning in blood to F0, we performed five biological replicates in GC1 (*N* = 34,233 offspring) and four biological replicates in GC2 (*N* = 21,978 offspring). We completed four biological replicates for supplemented F1 (GC1 = 26,376 offspring; GC2 = 17,079 offspring) and three biological replicates for unsupplemented F1 (GC1 = 18,876 offspring; GC2 = 9,689 offspring).

### Assessing effects of Pan supplementation via water on reproduction of older* A. stephensi*

For these experiments, 120 female mosquitoes were housed in 2-L polypropylene containers. These mosquitoes were provisioned with Pan via water at 11, 12, and 13 days post-adult eclosion. On day 14, they were offered an unsupplemented blood meal as described above. As we found anecdotally that older mosquitoes required additional time for oogenesis, females were moved to individual housing at 48 h postfeeding for oviposition. These older females were allowed 72 h for oviposition then transferred back to group housing. The number of females that oviposited and the number of eggs in each tube were counted and recorded for each treatment group. Three biological replicates were performed using separate cohorts of *A. stephensi* (*N* = 310 females).

### Pantothenate quantification in mosquito tissues

**Fig. 2:**
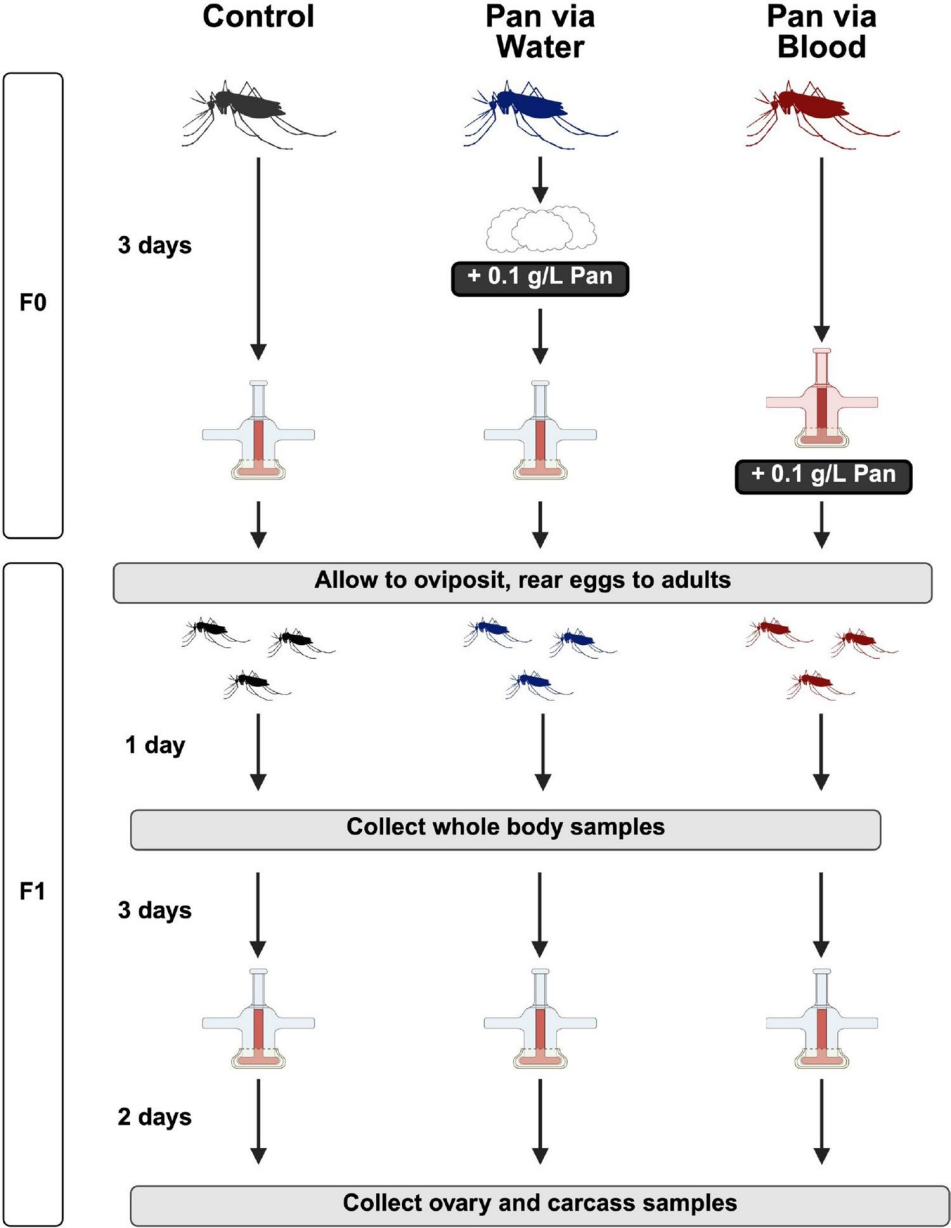
Sample preparation for the assessment of Pan levels in unsupplemented F1 offspring of Pan-supplemented F0 females. Adult females (F0) were provided Pan-supplemented water, Pan-supplemented blood, or no Pan. Following a blood meal, eggs were reared to F1 adults. Whole bodies of F1 females were collected at 1 day post-adult eclosion. F1 females from all groups were provided an unsupplemented blood meal at 4 days post adult eclosion, and 48 h later the ovaries were separated from the carcass of bloodfed females

To evaluate the Pan levels in whole bodies, dissected ovaries and remaining carcasses (whole bodies minus ovaries), adult female mosquitoes were provisioned with Pan via water or blood or received no Pan (Fig. 2). These females were individually housed to maintain consistency with mosquitoes used in other experiments. Eggs produced by these mosquitoes were reared into F1 adults. Whole bodies of F1 females were collected at 1 day post-adult eclosion. At 4 days post-adult eclosion, F1 females from each group were provided an unsupplemented blood meal. Any nonfed females were removed. Developing ovaries from bloodfed F1 females were dissected for collection with remaining carcasses at 48 h post-blood meal. Two biological replicates were performed with 60 F0 females per treatment group per replicate. For F1 whole body measurements, five whole bodies were pooled per sample (*N* = 180 females). For F1 carcass and ovary measurements, five carcasses were pooled per sample (*N* = 90 females) and five pairs of ovaries (*N* = 90 females) were pooled per sample.

To assess Pan levels in the samples above, we utilized the Pan assay in foodstuffs from Association of Official Analytical Chemists (AOAC) International [34], which we previously optimized [13] and validated with mass spectrometry (Supplementary Material Fig. S1) as described [36]. Briefly, whole mosquitoes or mosquito tissues were homogenized in 200 µl of homogenization buffer [100 mM acetate buffer (pH 4.6) papain (20 mM), α-amylase (8.5 mM), 2% chloroform]. Samples were then incubated overnight at 37 °C and autoclaved. The prepared Pan samples and a standard curve of known Pan concentrations were added to Pan-deficient media containing *Lactobacillus plantarum* [American Type Culture Collection (ATCC) 8014], which requires Pan for growth. *L. plantarum* growth was assessed after a 20 h incubation at 37 °C by measuring turbidity at OD550 and comparing the unknown mosquito samples against the Pan standard curve. Assays were replicated at least three times with separate cohorts of *A. stephensi*.

### Statistical analyses

Differences in blood feeding tendency, oviposition, and sex ratio of offspring among treatment groups and gonotrophic cycles were analyzed by the chi-squared test. The effects of treatment on the average number of eggs per female when there were three or more treatments were analyzed via one-way analysis of variance (ANOVA). For comparisons between gonotrophic cycles or when there were two treatment groups, the average number of eggs per female was analyzed via unpaired *t*-test. Outliers in clutch size data were detected using Robust regression and Outlier (ROUT) and removed from further analyses. Pan levels were analyzed by ANOVA and Tukey’s test. Analyses were performed with GraphPad Prism version 10.3.1 (GraphPad Software, Boston, MA, USA).

## Results

### Pan effects on blood feeding tendency differed based on supplementation scheme, with transgenerational effects for both water and blood delivery

**Fig. 3:**
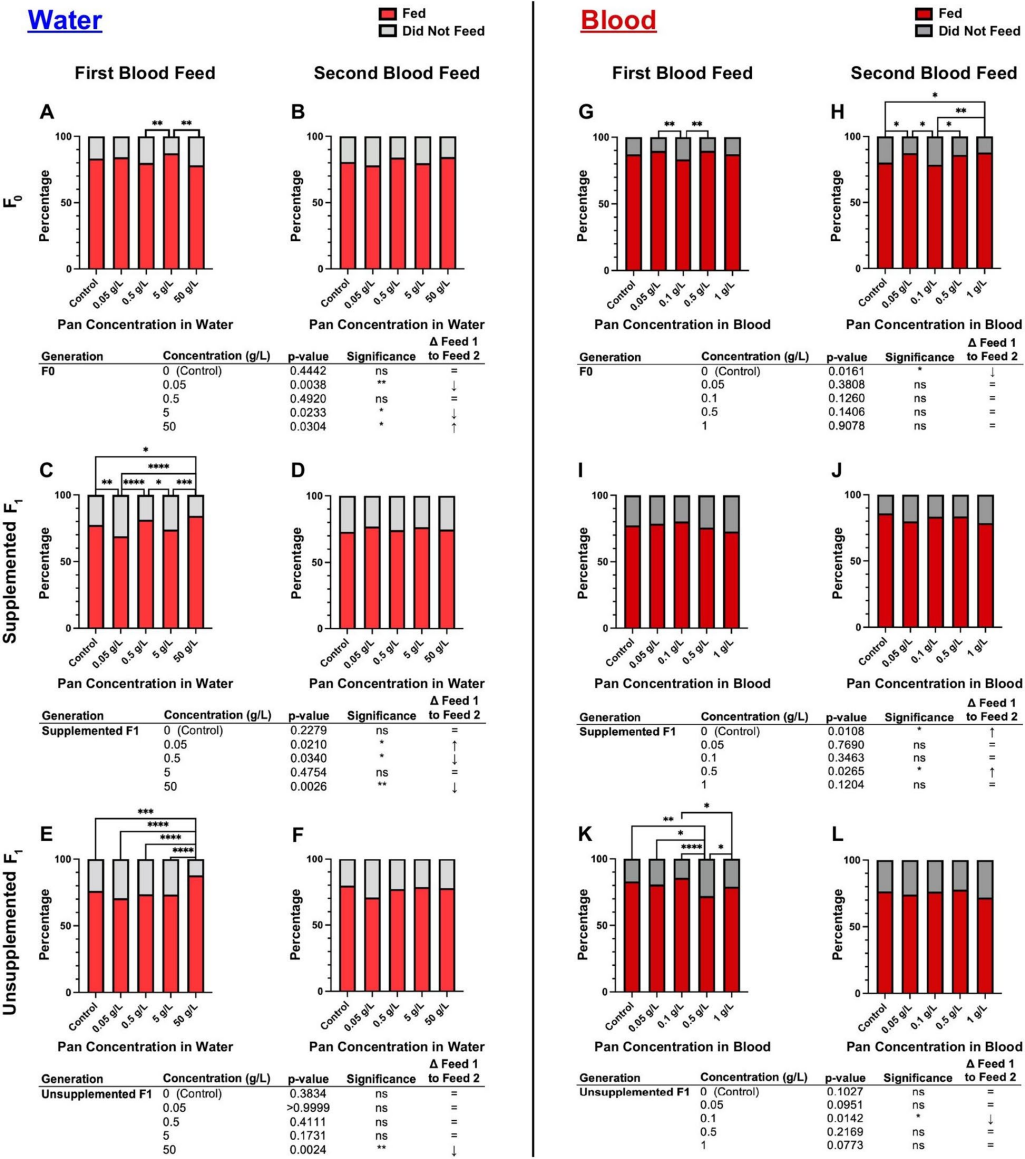
Pan effects on blood feeding tendency differed on the basis of the supplementation scheme, with transgenerational effects for both water and blood delivery. Left (**A**–**F**): percentages of females that fed or did not feed following 3 days of Pan supplementation via water. Right (**G**–**L**): percentages of females that fed or did not feed following Pan supplementation via blood. Results are shown for the first blood meal (**A**,**C**,**E**,**G**,**I**,**K**) and second blood meal (**B**,**D**,**F**,**H**,**J**,**L**). Tables show comparisons of blood feeding tendency between first and second blood meals within the same treatment group. Water supplementation: F0 *N* = 6 replicates, supplemented F1 *N* = 4 replicates, unsupplemented F1 *N* = 3 replicates. Blood supplementation: F0 first blood feed = 5 replicates, F0 second blood feed = 4 replicates, supplemented F1 = 4 replicates, unsupplemented F1 = 3 replicates. Data were analyzed by chi-squared test (*α* = 0.05). *P < 0.05, **P < 0.01, ***P < 0.001, ****P < 0.0001

Pan supplementation via water had no effect on the tendency to consume a blood meal in F0 mosquitoes (Fig. 3A, B; Supplementary Material Table S1). In F1 offspring, effects on blood feeding tendency varied with concentration. Specifically, supplemented F1 provisioned with Pan exhibited significant differences in their tendency to take a first blood meal (Fig. 3C), with differences between treatment groups varying by concentration. There were, however, no significant effects on supplemented F1 tendency to take a second blood meal (Fig. 3D). Unsupplemented F1 offspring of F0 mosquitoes supplemented with 50 g/L Pan were significantly more likely to take a first blood meal compared with all other groups (Fig. 3E), but there were no effects on tendency to take a second blood meal (Fig. 3F).

Pan provisioning via blood meal was associated with blood feeding patterns that were distinct from those associated with provisioning by water. In contrast to Pan supplementation in water, F0 females provisioned with Pan via blood meal exhibited concentration-dependent differences in tendency to take first and second blood meals (compare Fig. 3A, B with Fig. 3G, H; Supplementary Material Table S1), with increased tendency to take a second blood meal in females supplemented with 0.05 or 1 g/L Pan relative to controls (Fig. 3H). Notably, F0 females that were provisioned with 0.1 g/L Pan were less likely to take first and second blood meals compared with other treatment groups (Fig. 3G,H). Additionally, in contrast to Pan supplementation in water, there were no differences among supplemented F1 offspring in tendency to take first or second blood meals (compare Fig. 3C,D with Fig. 3I,J). However, unsupplemented F1 offspring from both water-supplemented mothers (Fig. 3E,F) and blood-supplemented mothers (Fig. 3K,L) exhibited differences in tendency to take a first blood meal, but not a second blood meal, with F1 offspring of females supplemented with 0.5 g/L less likely to feed than all other groups (Fig. 3K). Notably, these results revealed transgenerational effects of Pan supplementation via both water and blood in that both sets of unsupplemented F1 offspring from supplemented mothers exhibited differences across groups in tendency to take a first blood meal (Fig. 3E,K).

To explore these differences further, we compared feeding tendency within a single treatment and filial group across first and second blood meals (e.g., control F0 tendency to take a first blood meal versus a second blood meal). When Pan was provisioned via water (Fig. 3A–F), significant differences within the F0 group and within each F1 group were associated with higher and lower feeding tendencies by treatment between the first and second blood meals. Notably, we saw a significant decrease in tendency to take a second blood meal compared with a first in both supplemented and unsupplemented F1 offspring of mothers provisioned with 50 g/L Pan (Fig. 3C–F). When Pan was provisioned via blood meal (Fig. 3G–L), F0 females that received Pan exhibited no difference in feeding tendency, whereas controls were less likely to take a second blood meal. Significant differences in supplemented F1 offspring were associated with increased feeding in the second versus first blood meals (Fig. 3I and J), while unsupplemented F1s from mother supplemented with 0.1 g/L Pan exhibited reduced feeding in the second versus first blood meal (Fig. 3K and L).

### Pan effects on oviposition differed on the basis of supplementation scheme, with transgenerational effects only for water delivery and within generation effects only for blood delivery

Our previous work showed that pantazine provisioning to young (3–5-day-old) *A. stephensi* decreased Pan and increased CoA levels [6], but there were no effects on egg production in these young females [13]. In subsequent work, however, we showed that Pan is allocated to *A. stephensi* ovaries as eggs develop, that knockdown of the putative *A. stephensi* Pan transporter significantly reduced follicle development, and that Pan supplementation in blood restored the reproductive output of 14-day-old *A. stephensi* [14]. Together, these data suggested that Pan is an important and potentially limiting nutrient for mosquito reproduction.

To assess the impacts of Pan supplementation on oviposition, we individually housed bloodfed F0 mosquitoes and their supplemented or unsupplemented F1 offspring to determine egg production per female. Females completed two reproductive cycles following provisioning of Pan via water or blood, as previously described.

**Fig. 4:**
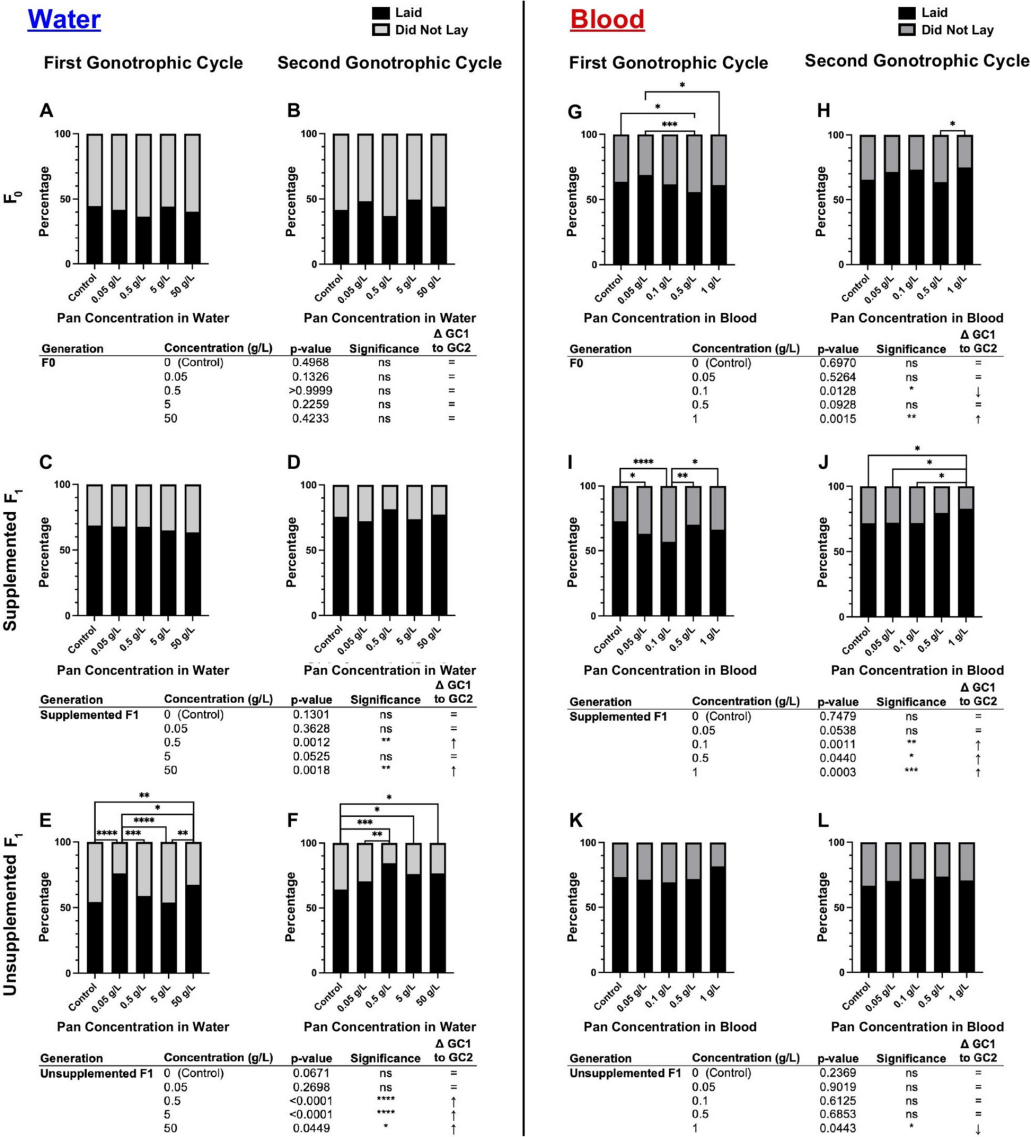
Pan effects on oviposition differ with supplementation method. Left (**A**–**F**): percentages of females that laid or did not lay eggs following 3 days of Pan supplementation via water and an unsupplemented blood meal. Right (**G**–**L**): percentages of females that laid or did not lay eggs following a Pan-supplemented blood meal. Results are shown for the first gonotrophic cycle (GC1; **A**,**C**,**E**,**G**,**I**,**K**) and second gonotrophic cycle (GC2; **B**,**D**,**F**,**H**,**J**,**L**). Tables show comparisons of oviposition percentages between GC1 and GC2 within the same treatment group. Water supplementation: F0 *N* = 6 replicates, supplemented F1 *N* = 4 replicates, unsupplemented F1 *N* = 3 replicates. Blood supplementation: F0 GC1 = 5 replicates, F0 GC2 = 4 replicates, supplemented F1 = 4 replicates, unsupplemented F1 = 3 replicates. Data were analyzed by chi-squared test (*α* = 0.05). *P < 0.05, **P < 0.01, ***P < 0.001, ****P < 0.0001

When Pan was supplemented via water (Fig. 4A–F; Supplementary Material Table S2), effects on oviposition were limited to unsupplemented F1 offspring (Fig. 4E,F), which were equally or more likely to oviposit across the first (GC1) and second (GC2) gonotrophic cycles relative to F1s from control mothers (Fig. 4E,F). By contrast, when Pan was supplemented via blood (Fig. 4G–L; Supplementary Material Table S2), significant effects on oviposition were observed in both F0s (Fig. 4G,H) and supplemented F1 offspring (Fig. 4I,J) but not in unsupplemented F1 offspring (Fig. 4K,L). In F0 females (Fig. 4G,H), these effects varied with treatment and gonotrophic cycle. In supplemented F1 offspring, however, females supplemented with 0.05 g/L and 0.1 g/L Pan were significantly less likely to oviposit than controls in GC1 (Fig. 4I). In GC2, however, F1 offspring supplemented with 1 g/L Pan were more likely than other groups to oviposit (Fig. 4J).

To explore these differences further, we compared oviposition within a single treatment and filial group across GC1 and GC2 (e.g., control F0 oviposition in GC1 versus GC2). In *A. stephensi* supplemented with Pan via water (Fig. 4A-F), no F0 treatment groups exhibited differences in oviposition between GC1 and GC2 (Figs. 4A and B). However, both supplemented and unsupplemented F1s of F0s that received higher concentrations of Pan in water showed increased oviposition in GC2 compared with GC1 (Fig. 4C–F). In females supplemented with Pan via blood meal (Figs. 4G–L), effects on F0 groups were variable with two groups showing decreased and increased oviposition relative to control (Fig. 4G and H). Supplemented F1 offspring, however, were significantly more likely to oviposit in GC2 compared with GC1 for the three highest concentrations of Pan supplementation (Fig. 4I and J), whereas effects on unsupplemented F1 offspring were limited to decreased oviposition in GC1 versus GC2 at the highest Pan dose provided to F0 mothers (Fig. 4K and L).

Taken together, when Pan was provisioned in water, only unsupplemented F1 offspring showed increased oviposition in GC1 and GC2 compared with control (Fig. 4E,F). These data suggest that F0 supplementation with Pan in water is associated only with transgenerational effects on *A. stephensi* reproduction. By contrast, Pan provisioning via blood meal was associated with effects on oviposition only during supplementation (Fig. 4G–J), with no transgenerational effects on unsupplemented F1 offspring (Fig. 4K,L).

### Pan supplementation altered clutch sizes within generations and transgenerationally, with universal increases between GC1 and GC2 in unsupplemented F1s from water-supplemented mothers

**Fig. 5:**
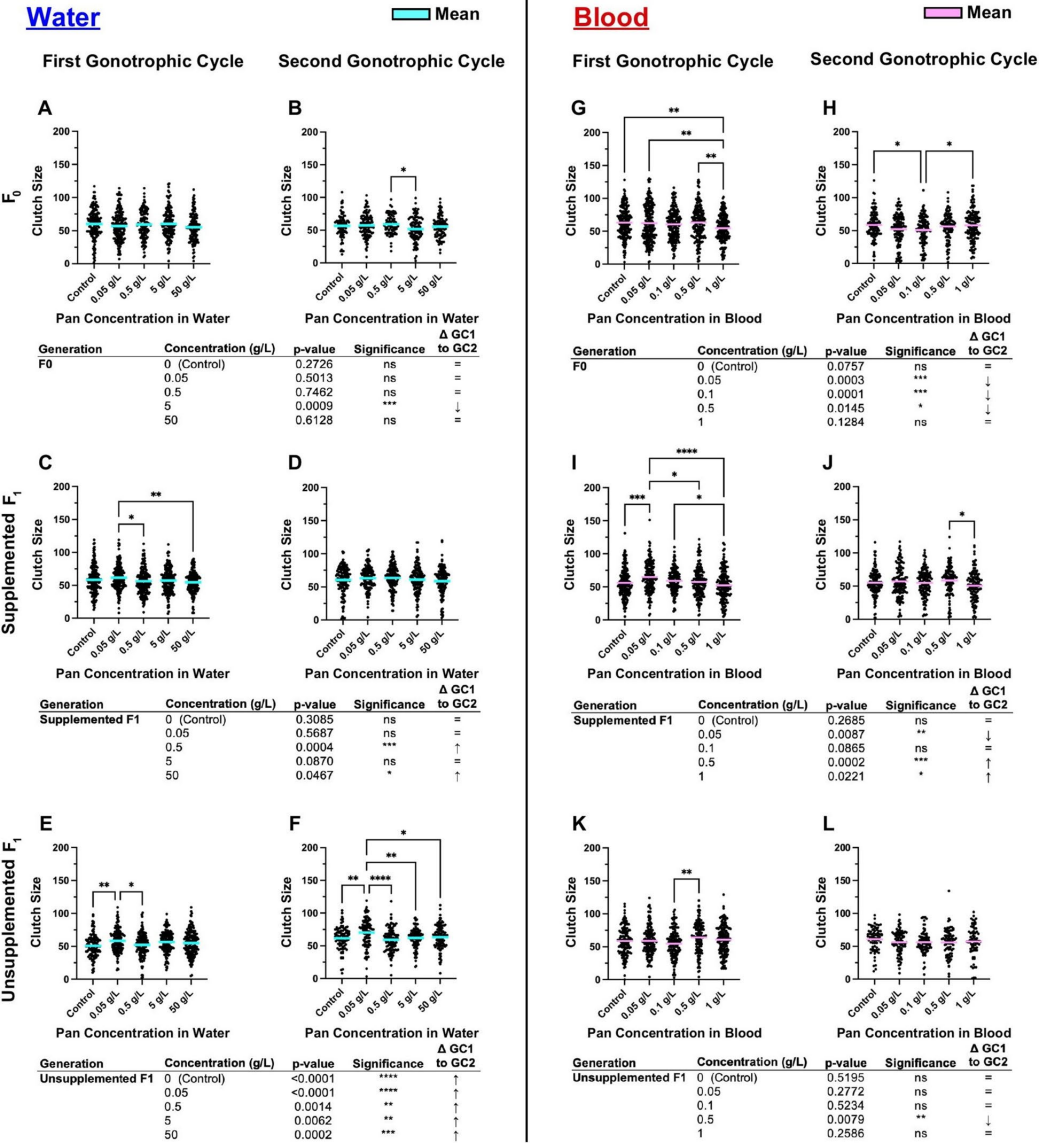
Pan supplementation altered clutch sizes within generations and transgenerationally based on method. Left (**A**-**F**): the number of eggs per female following 3 days of Pan supplementation via water and an unsupplemented blood meal. Right (**G**–**L**): the number of eggs per female following a Pan supplemented blood meal. Results are shown for the first gonotrophic cycle (GC1; **A**,**C**,**E**,**G**,**I**,**K**) and second gonotrophic cycles (GC2; **B**,**D**,**F**,**H**,**J**,**L**). Tables show comparisons of clutch size between GC1 and GC2 within the same treatment group. Water supplementation: F0 *N* = 6 replicates, supplemented F1 *N* = 4 replicates, unsupplemented F1 *N* = 3 replicates. Blood supplementation: F0 GC1 = 5 replicates, F0 GC2 = 4 replicates, supplemented F1 = 4 replicates, unsupplemented F1 = 3 replicates. Data were analyzed by one-way ANOVA (α = 0.05). *P < 0.05, **P < 0.01, ***P < 0.001, ****P < 0.0001

When Pan was supplemented via water (Fig. 5A–F; Supplementary Material Tables S3,S4), effects were noted in supplemented mothers in GC2 (Fig. 5B), in supplemented F1 offspring in GC1 (Fig. 5C) and unsupplemented F1 offspring in GC1 and GC2 (Fig. 5E,F). In particular, the supplemented and unsupplemented offspring of mothers supplemented with 0.05 g/L Pan had the greatest mean clutch sizes in these cycles. Increased oviposition in unsupplemented F1 offspring from mothers supplemented with 0.05 g/L Pan (Fig. 4E) was also consistent with larger clutch sizes in this group in both GC1 and GC2 (Fig. 5E,F).

When Pan was supplemented via blood meal (Fig. 5G–L; Supplementary Material Tables S3,S4), significant effects were observed during supplementation of F0s (Fig. 5G,H) and supplemented F1 offspring (Figs. 5I,J), with a single difference in clutch size noted in unsupplemented F1 offspring in GC1 (Fig. 5K). The latter was reflective of the lack of differences in oviposition in GC1 and GC2 in this group (Fig. 4K,L). Within groups, F0s supplemented with 1 g/L Pan had a significantly smaller mean clutch size compared with control in GC1 (Fig. 5G), while in GC2, F0s supplemented with 0.1 g/L Pan had a significantly smaller mean clutch size than the control (Fig. 5H). In the offspring of blood-supplemented females, mean clutch size was highest in GC1 in F1 supplemented females provisioned with 0.05 g/L Pan (Fig. 5I), but this pattern was not observed in GC2 (Fig. 5J).

To explore these differences further, we compared clutch sizes within a single treatment and filial group across GC1 and GC2 (e.g., control F0 clutch size in GC1 versus GC2). When Pan was provisioned in water (Fig. 5A–F), a single decrease in F0 clutch size in GC1 versus GC2 was noted for one treatment group (Fig. 5A,B). This contrasted with increased clutch sizes in two treatment groups for supplemented F1s and increased clutch sizes for all unsupplemented F1 groups between GC1 and GC2 (Fig. 5C–F). When Pan was provisioned via blood meal (Fig. 5G–L), F0 clutch sizes were more frequently reduced by treatment compared with water supplementation (three F0 blood-supplemented groups in Fig. 5G,H versus one F0 water-supplemented group in Fig. 5A,B). Clutch size between GC1 and GC2 in blood-supplemented F1 offspring was decreased at the lowest level of Pan supplementation and increased at the two highest levels of supplementation (Fig. 5I, J). In contrast to universal increases in clutch sizes between GC1 and GC2 in unsupplemented F1 offspring of water-supplemented mothers (Fig. 5E,F), only a single treatment (0.5 g/L) delivered via blood meal to F0s was associated with a change in unsupplemented F1 clutch size, a decrease between GC1 and GC2 (Fig. 5K, L).

### Pan supplementation minimally altered offspring sex ratio

**Fig. 6:**
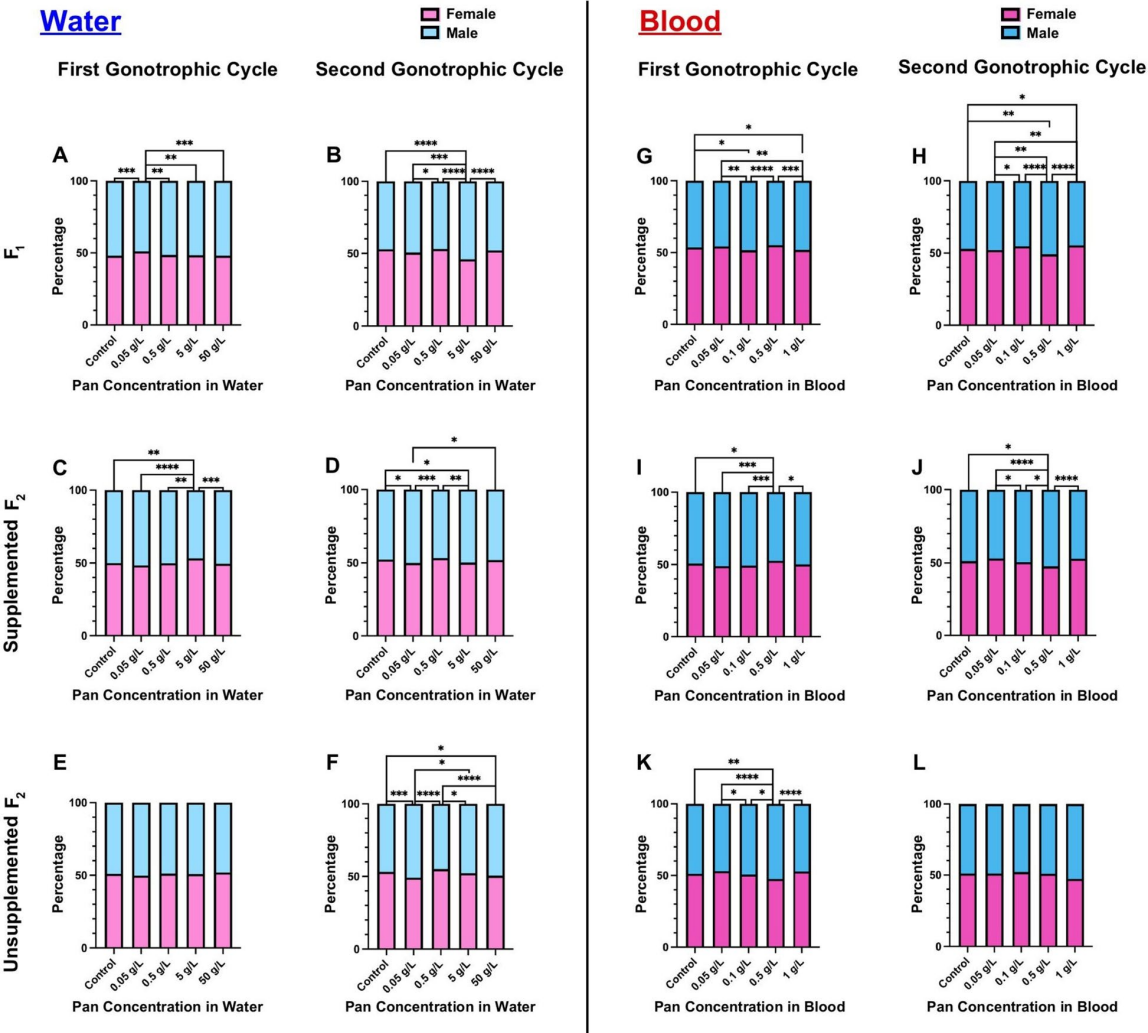
Pan supplementation minimally altered offspring sex ratio. Left (**A**-**F**): the percentages of female and male offspring following 3 days of Pan supplementation via water and an unsupplemented blood meal. Right (**G**-**L**): the percentages of male and female offspring following a Pan-supplemented blood meal. Results are shown for the first gonotrophic cycle (**A**,**C**,**E**,**G**,**I**,**K**) and second gonotrophic cycle (**B**,**D**,**F**,**H**,**J**,**L**). Water supplementation: F0 *N* = 6 replicates, supplemented F1 *N* = 4 replicates, unsupplemented F1 *N* = 3 replicates. Blood supplementation: F0 GC1 = 5 replicates, F0 GC2 = 4 replicates, supplemented F1 = 4 replicates, unsupplemented F1 = 3 replicates. Data were analyzed by chi-squared test (α = 0.05). *P < 0.05, **P < 0.01, ***P < 0.001, ****P < 0.0001

In several vertebrate and invertebrate species, alterations to endogenous Pan levels have been shown to impact offspring sex [21, 25, 26]. To determine whether Pan supplementation altered sex ratio in *A. stephensi*, we counted female and male offspring from each of our treatment groups. Given the large sample sizes of males and females across our replicates, several significant differences were observed, but deviations from the expected 50:50 sex ratio were small (Fig. 6A–L; Supplementary Material Table S5).

### Pan supplementation via water did not alter fecundity of 14-day-old females

We showed previously that Pan levels in female *A. stephensi* declined by more than 50% by 14 days post-adult eclosion [14]. Further, Pan provisioning via a blood meal reversed significant declines in oviposition and clutch size in 14-day-old *A. stephensi* females to levels observed in 3–5-day-old females [14]. In this study, Pan provisioning via water was associated with transgenerational effects, increasing oviposition and clutch size in unsupplemented F1 females (Figs. 4E,F and 5E,F). By GC2, females in these experiments were over 14 days old, suggesting that Pan provisioning of 14-day-old females via water might recover the loss in fecundity associated with aging or achieve increased fecundity similar to that observed in unsupplemented F1 offspring of water-supplemented mothers.

**Fig. 7:**
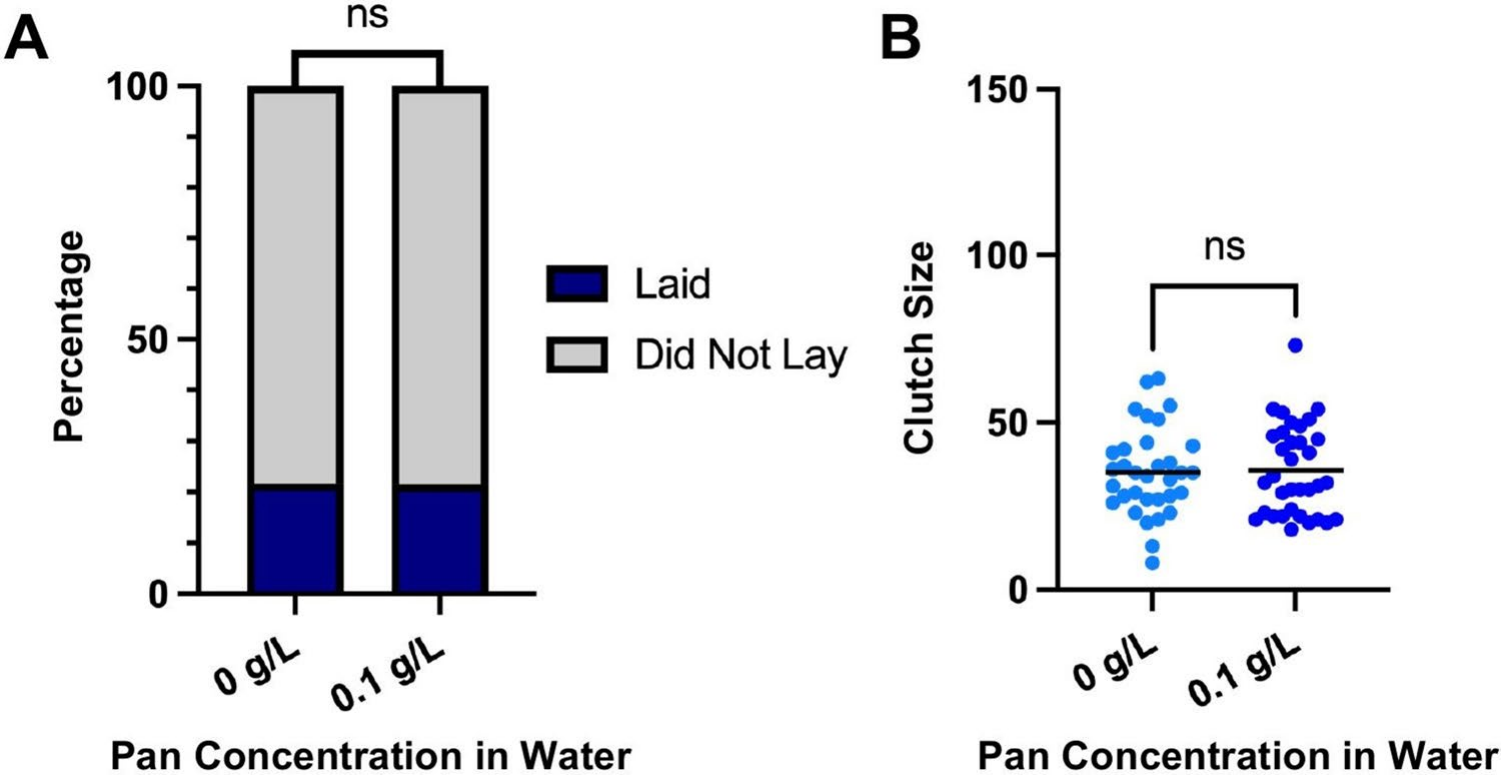
Pan supplementation via water did not alter fecundity of 14-day-old females. **A** The percentages of females that laid or did not lay. Data were analyzed by chi-squared test (*α* = 0.05). **B** Numbers of eggs laid per female. Data were analyzed by two-tailed *t*-test (*α* = 0.05)

In contrast to our expectations, we observed no significant changes to oviposition or clutch size in water-supplemented 14-day-old females (Fig. 7A,B). Accordingly, the transgenerational effects of Pan provisioning to 3–5-day-old females that are 14 days old at the time of observed increases in fecundity cannot be replicated by provisioning Pan via water directly to 14-day-old females. As noted, delivery of Pan in blood to 14-day-old females recovered fecundity to levels observed in 3–5-day-old females [14]. Taken together, the effects of Pan supplementation observed in our current study are transgenerational at 14 days post-adult eclosion, while the previously observed effect of blood delivery of Pan to 14-day-old females occurred within the reproductive cycle of those supplemented females [14].

### Ovary Pan levels in unsupplemented F1 offspring from water-supplemented mothers reflected transgenerational effects on oviposition and clutch size

**Fig. 8:**
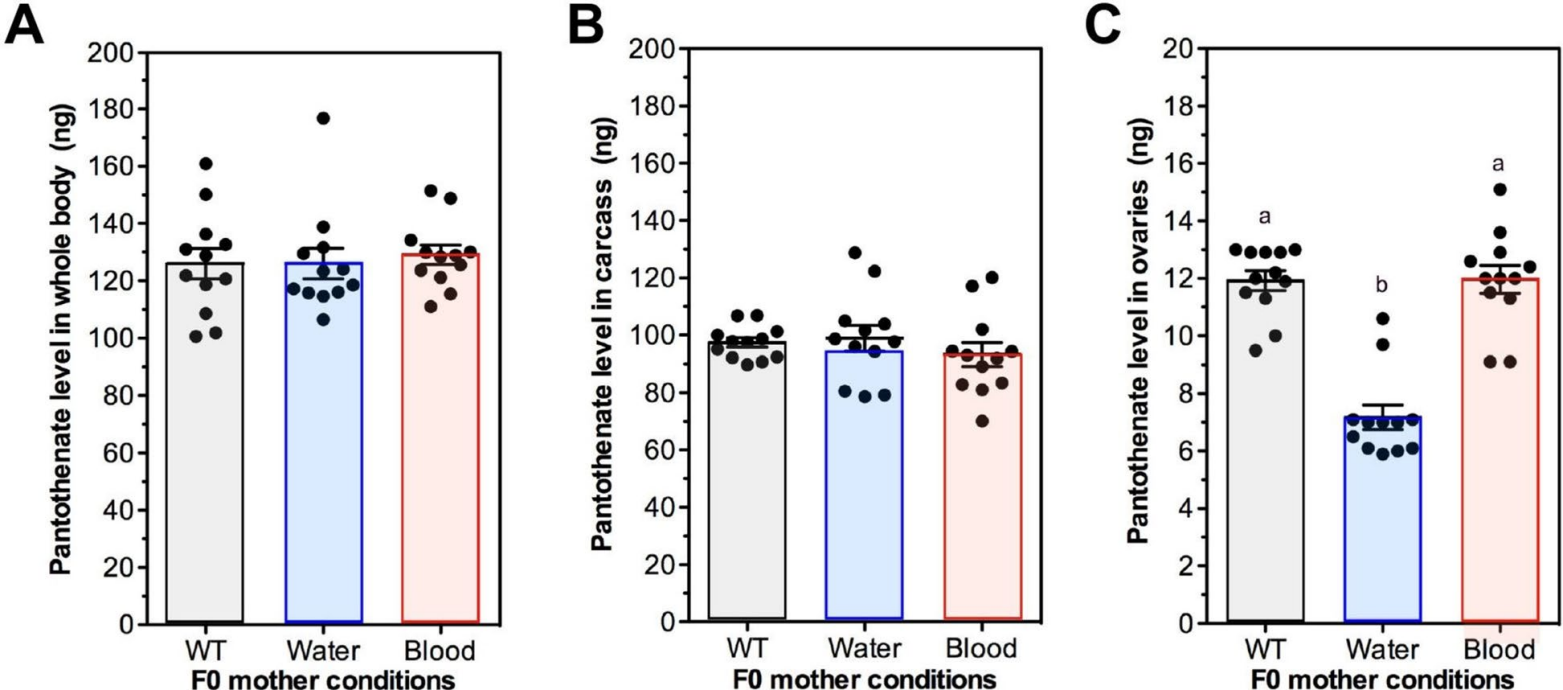
Ovary Pan levels in unsupplemented F1 offspring from water-supplemented mothers were reduced relative to levels in control (WT) and blood-supplemented mothers. Data were collected from tissues from two separate *A. stephensi* cohorts prepared as shown in Fig. 2. **A** Whole body Pan levels of unsupplemented F1 females derived from F0 mothers not supplemented with Pan (WT) or supplemented with 0.1 g/L Pan in water or in blood. **B** Carcass Pan levels in unsupplemented F1 females as in (**A**). (**C**) Ovary Pan levels in F1 unsupplemented F1 females as in (**A**). Data were analyzed by ANOVA and Tukey’s test (α = 0.05). Different lowercase letters above bars indicate significant differences between groups

Given the association between F0 water supplementation and transgenerational effects on unsupplemented F1 oviposition (Fig. 4E,F) and clutch size (Fig. 5E,F), we sought to determine whether Pan levels in these unsupplemented offspring were reflective of the F0 supplementation scheme (Fig. 2). In combined data from replicated studies, there were no differences in Pan levels in whole bodies or carcasses of unsupplemented F1 offspring from control mothers or mothers supplemented in water or blood (Fig. 8A,B). However, Pan levels in ovaries from unsupplemented F1 offspring were significantly lower than ovary levels from F1 unsupplemented offspring derived from control mothers or mothers supplemented via blood (Fig. 8C). Further, ovary Pan levels in unsupplemented F1 offspring from blood-supplemented mothers did not differ from control levels (Fig. 8C), a pattern reflective of the lack of transgenerational effects on oviposition of unsupplemented F1 offspring from blood-supplemented mothers (Fig. 4K,L). Hence, altered ovary Pan levels were specifically associated with increased fecundity of unsupplemented F1 offspring from water-supplemented mothers.

## Discussion

Pan and its biochemical products are critical to many eukaryotic cellular functions and impact a broad range of life history traits in other species [21–26]. The manipulation of mosquito Pan reserves through PanK has been identified as a viable target for novel transmission-blocking interventions for malaria [5–7]. Accordingly, a more complete understanding of the roles of Pan in *Anopheles* spp. is important to determine the broader potential of this strategy and to identify new vector control strategies for malaria. Adult mosquitoes acquire key nutrients from both nectar and blood, and the nutritional composition of each impacts a variety of traits important for vectorial capacity including reproduction and feeding behaviors [17, 18, 36]. In these studies, we investigated the effects of Pan supplementation via water, which, similar to nectar, is directed to the crop then slowly released into the midgut, and blood, which transits directly to the midgut for digestion, on the tendency of female mosquitoes to take a blood meal, oviposition behavior, clutch size, and offspring sex ratio in *A. stephensi*. Our results showed that the outcomes of Pan provisioning are influenced by concentration, timing, delivery method, and parental supplementation status. These effects were also distinct from those associated with decreased Pan availability or the inhibition of CoA biosynthesis [13]. Notably, Pan supplementation effects were, in several cases, transgenerational, occurring in the absence of further supplementation.

Blood feeding tendency was influenced by Pan supplementation but did not follow a dose-dependent pattern (Fig. 3). Supplementation of Pan via water impacted consumption of the first blood meal in both supplemented and unsupplemented F1 offspring, with offspring supplemented with 50 g/L Pan more likely to take an initial blood meal (Fig. 3C,E). Pan supplementation via blood increased feeding in both F0 and their unsupplemented F1 offspring (Fig. 3G,H,K). Previous studies in the honeybee demonstrated that provisioning of high Pan concentrations (71.4 g/L) to *Apis mellifera* significantly increased their energy consumption [22]. Given that excess CoA and acetyl-CoA can directly increase glycolysis in mammalian hepatocytes through mitochondrial lysine acetylation [37], increased energy consumption in Pan-supplemented honeybees could reflect a need to fuel enhanced glycolysis. As blood is an essential energy source for female mosquitoes, similar effects of Pan on cellular biochemistry in *A. stephensi* could underlie differences in feeding tendency.

We found that the effects of Pan on fecundity depended on the method of supplementation. When delivered in water, Pan had no within-generation effects on oviposition (Fig. 4A–D). Rather, effects on oviposition were observed only in unsupplemented F1 offspring (Fig. 4E,F), which generally exhibited a greater tendency to oviposit relative to control across GC1 and GC2. Larger clutch sizes were observed in these offspring from mothers provisioned with 0.05 g/L Pan (Fig. 5E,F). These observations suggested that Pan acquired in water by young female *A. stephensi*—as the offspring were the product of 3–5-day-old females provisioned with Pan—drives transgenerational effects. Pan supplementation via blood was associated with effects on oviposition and clutch size relative to controls within F0s and in supplemented F1 offspring (Fig. 4G,I,J and Fig. 5G,H,I). There were no effects on oviposition and clutch size relative to controls in unsupplemented F1 offspring (Fig. 4K,L and Fig. 5K,L), suggesting that nutrient context strongly influences the effects of ingested Pan. When Pan was supplemented via water to older, Pan-depleted females, there was no effect on oviposition tendency or clutch size (Fig. 7). This contrasted with our previous results, where Pan supplementation via a blood meal increased both parameters [14], suggesting that in the absence of other nutrients, Pan may not be sufficient to counteract age-related declines in fecundity. In further studies here, we demonstrated a specific association between reduced ovary Pan levels and increased fecundity in unsupplemented offspring from young, water-supplemented mothers, highlighting transgenerational effects on F1 fecundity resulting from F0 water supplementation. Collectively, our data suggest that Pan effects on fecundity of F0 and F1 *A. stephensi* reflect an interaction between mosquito age, parental nutrition, and supplementation via water or blood.

One possible explanation for transgenerational effects of Pan supplementation is maternal provisioning of CoA and precursors to developing ovarian follicles. This has been documented in *D. melanogaster*, where maternally derived CoA and precursors support early larval development in the absence of alternative Pan or CoA sources [31]. Similarly, mosquito nutritional status, including the consumption of plant nectar, has been shown to impact gene expression, fecundity, and lipid provisioning to developing eggs [17, 28, 38]. Taken together with our previous data showing that Pan is essential for follicle development in *A. stephensi* and that Pan levels decline with age [14], we infer that maternally provisioned CoA and precursors from blood and nectar in *A. stephensi* contribute to mosquito reproductive success likely through multiple mechanisms.

Nutrient availability has also been linked to numerous epigenetic modifications [39]. In human tissues, Pan treatment has been shown to increase PanK expression in a dose-dependent manner [40]. PanK is the rate-limiting step of CoA biosynthesis, and increased PanK activity has been demonstrated to increase CoA levels in *A. stephensi* [13]. Acetyl-CoA derived from CoA is utilized in the acetylation of proteins, including histones, with the levels of CoA and acetyl-CoA correlated with levels of histone acetylation [32]. Accordingly, Pan supplementation may induce epigenetic modifications in *A. stephensi* via increased acetyl-CoA availability. Further studies are needed to determine whether Pan supplementation indeed increases histone and non-histone protein modifications in *A. stephensi*.

While these studies provide insights into the effects of Pan on *A. stephensi* physiology, the limitations of this work should be considered. We evaluated blood feeding, oviposition, clutch size and offspring sex ratio. Factors such as host-seeking behavior, environmental tolerance, and fitness traits in F1 mosquitoes and further generations should be evaluated in future studies. F1 females were reared only from GC1 of supplemented mothers, so the effects on offspring from GC2 were not defined in our studies. Additionally, supplementation via water fails to capture the complexity of the composition of plant nectars. These bioactive solutions and their associated microbiota are likely important to the effects of ingested Pan under natural conditions. We also lack direct insight into the intracellular fate of Pan. Given that excess Pan can increase CoA synthesis, it is likely critical to understand the tissue localization, persistence, and metabolism of CoA and its products in *A. stephensi*.

## Conclusions

Our data affirm that Pan is utilized for *A. stephensi* reproduction and, therefore, restricting Pan bioavailability could be a viable target for mosquito population control. Our previous work also showed that reducing Pan bioavailability by shifting Pan to CoA limits malaria parasite development in *A. stephensi* [6]. This raises the question as to whether a single manipulation—converting Pan to CoA in female *A. stephensi*—could reduce mosquito reproduction and parasite infection, additively reducing malaria parasite transmission. Multiple questions remain, however, regarding the mechanism by which Pan supplementation produces transgenerational effects. Manipulation of Pan to CoA conversion as a control strategy—perhaps via delivery of pantazines via attractive sugar bait stations—will need to account for differences in age and nutritional status of mosquitoes in the field. With these questions in mind, our data provide an important foundation for future studies on the roles of Pan and CoA in *Anopheles* spp. as well as potential benefits and risks of interventions that target the Pan to CoA biosynthesis pathway for malaria control.

## Supplementary Information

Additional file 1.

## Data Availability

Data supporting the main conclusions of this study are included in the manuscript.

## Abbreviations

CoA: Coenzyme A
F0: Zero filial, parental generation
F1: First filial, first generation of offspring
F2: Second filial, second generation of offspring
GC1: First gonotrophic cycle
GC2: Second gonotrophic cycle
Pan: Pantothenate
