# Supplementary material for: Pantothenate regulates feeding and reproduction in the malaria vector Anopheles stephensi, with patterns dependent on supplementation scheme and parental nutrition
Source: Parasit Vectors. 2025 Aug 4;18:334. doi: 10.1186/s13071-025-06959-w (PMC12323043; doi:10.1186/s13071-025-06959-w)
Supplement: Supplementary file 1 — Additional file 1. [file 13071_2025_6959_MOESM1_ESM.docx]

**
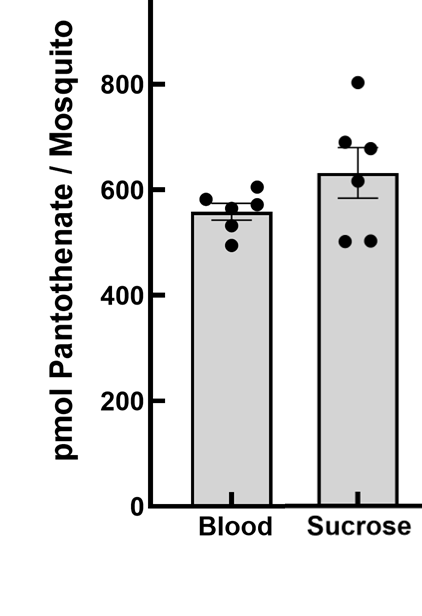
Supplementary Figure 1. Endogenous Pan levels in the whole bodies of control adult female *A. stephensi* fed blood or sugar as determined by liquid chromatography-mass spectrometry (LC-MS).** Female *A. stephensi* mosquitoes were maintained on 10% sucrose ad libitum. At three days post-eclosion a subset of mosquitoes were provisioned with blood. Six pools of blood or sugar fed mosquitoes (n=5 mosquitoes/pool) were homogenized in 80% methanol and endogenous Pan levels determined using LC-MS as previously described (Sharma et. al., 2018). Pan was measured using ^13^C Pan as the internal standard. Endogenous Pan levels in whole female mosquitoes were consistent between the LC-MS and bacterial assays (560 pmol/mosquito; Isoe et.al., 2025).

**Supplementary Table 1.** Differences in tendency to blood feed between *A. stephensi* supplemented with Pan via water or blood. Data were analyzed by Chi-Square test (α = 0.05). * p < 0.05, ** p < 0.01, *** p < 0.001, **** p < 0.0001.

| **Pan Source** | **Generation** | **Gonotrophic Cycle** | **Treatment 1 (g/L)** | **Treatment 2 (g/L)** | **p-value** | **Significance** |
| --- | --- | --- | --- | --- | --- | --- |
| **Water** | **F0** | **1** | Overall |  | 0.0061 | ** |
|  |  |  | 0 (Control) | 0.05 | 0.6630 | ns |
|  |  |  | 0 (Control) | 0.5 | 0.1865 | ns |
|  |  |  | 0 (Control) | 5 | 0.0857 | ns |
|  |  |  | 0 (Control) | 50 | 0.0762 | ns |
|  |  |  | 0.05 | 0.5 | 0.0757 | ns |
|  |  |  | 0.05 | 5 | 0.1874 | ns |
|  |  |  | 0.05 | 50 | 0.0679 | ns |
|  |  |  | 0.5 | 5 | 0.0028 | ** |
|  |  |  | 0.5 | 50 | 0.5567 | ns |
|  |  |  | 5 | 50 | 0.0010 | *** |
|  |  | **2** | Overall |  | 0.1712 | ns |
|  | **F1 Supplemented** | **1** | Overall |  | <0.0001 | **** |
|  |  |  | 0 (Control) | 0.05 | 0.0075 | ** |
|  |  |  | 0 (Control) | 0.5 | 0.2111 | ns |
|  |  |  | 0 (Control) | 5 | 0.2581 | ns |
|  |  |  | 0 (Control) | 50 | 0.0149 | * |
|  |  |  | 0.05 | 0.5 | <0.0001 | **** |
|  |  |  | 0.05 | 5 | 0.1041 | ns |
|  |  |  | 0.05 | 50 | <0.0001 | **** |
|  |  |  | 0.5 | 5 | 0.0129 | * |
|  |  |  | 0.5 | 50 | 0.3030 | ns |
|  |  |  | 5 | 50 | 0.0002 | *** |
|  |  | **2** | Overall |  | 0.8337 | ns |
|  | **F1 Unsupplemented** | **1** | Overall |  | <0.0001 | **** |
|  |  |  | 0 (Control) | 0.05 | 0.1447 | ns |
|  |  |  | 0 (Control) | 0.5 | 0.4729 | ns |
|  |  |  | 0 (Control) | 5 | 0.4414 | ns |
|  |  |  | 0 (Control) | 50 | 0.0002 | *** |
|  |  |  | 0.05 | 0.5 | 0.4350 | ns |
|  |  |  | 0.05 | 5 | 0.4524 | ns |
|  |  |  | 0.05 | 50 | <0.0001 | **** |
|  |  |  | 0.5 | 5 | >0.9999 | ns |
|  |  |  | 0.5 | 50 | <0.0001 | **** |
|  |  |  | 5 | 50 | <0.0001 | **** |
|  |  | **2** | Overall |  | 0.1877 | ns |
| **Blood** | **F0** | **1** | Overall |  | 0.0122 | * |
|  |  |  | 0 (Control) | 0.05 | 0.2350 | ns |
|  |  |  | 0 (Control) | 0.1 | 0.1057 | ns |
|  |  |  | 0 (Control) | 0.5 | 0.2559 | ns |
|  |  |  | 0 (Control) | 1 | >0.9999 | ns |
|  |  |  | 0.05 | 0.1 | 0.0024 | ** |
|  |  |  | 0.05 | 0.5 | >0.9999 | ns |
|  |  |  | 0.05 | 1 | 0.2206 | ns |
|  |  |  | 0.1 | 0.5 | 0.0038 | ** |
|  |  |  | 0.1 | 1 | 0.0828 | ns |
|  |  |  | 0.5 | 1 | 0.2353 | ns |
|  |  | **2** | Overall |  | 0.0115 | * |
|  |  |  | 0 (Control) | 0.05 | 0.0360 | * |
|  |  |  | 0 (Control) | 0.1 | 0.7352 | ns |
|  |  |  | 0 (Control) | 0.5 | 0.095 | ns |
|  |  |  | 0 (Control) | 1 | 0.0268 | * |
|  |  |  | 0.05 | 0.1 | 0.0135 | * |
|  |  |  | 0.05 | 0.5 | 0.6879 | ns |
|  |  |  | 0.05 | 1 | 0.8905 | ns |
|  |  |  | 0.1 | 0.5 | 0.0419 | * |
|  |  |  | 0.1 | 1 | 0.0098 | ** |
|  |  |  | 0.5 | 1 | 0.5953 | ns |
|  | **F1 Supplemented** | **1** | Overall |  | 0.1081 | ns |
|  |  | **2** | Overall |  | 0.2277 | ns |
|  | **F1 Unsupplemented** | **1** | Overall |  | 0.0007 | *** |
|  |  |  | 0 (Control) | 0.05 | 0.5147 | ns |
|  |  |  | 0 (Control) | 0.1 | 0.4123 | ns |
|  |  |  | 0 (Control) | 0.5 | 0.0022 | ** |
|  |  |  | 0 (Control) | 1 | 0.2449 | ns |
|  |  |  | 0.05 | 0.1 | 0.1243 | ns |
|  |  |  | 0.05 | 0.5 | 0.0152 | * |
|  |  |  | 0.05 | 1 | 0.6891 | ns |
|  |  |  | 0.1 | 0.5 | <0.0001 | **** |
|  |  |  | 0.1 | 1 | 0.0334 | * |
|  |  |  | 0.5 | 1 | 0.0459 | * |
|  |  | **2** | Overall |  | 0.7323 | ns |

**Supplementary Table 2.** Differences in tendency to oviposit between *A. stephensi* supplemented with Pan via water or blood. Data were analyzed by Chi-Square test (α = 0.05). * p < 0.05, ** p < 0.01, *** p < 0.001, **** p < 0.0001.

| **Pan Source** | **Generation** | **Gonotrophic Cycle** | **Treatment 1 (g/L)** | **Treatment 2 (g/L)** | **p-value** | **Significance** |
| --- | --- | --- | --- | --- | --- | --- |
| **Water** | **F0** | **1** | Overall | | 0.2006 | ns |
|  |  | **2** | Overall | | 0.0580 | ns |
|  | **F1 Supplemented** | **1** | Overall | | 0.6241 | ns |
|  |  | **2** | Overall | | 0.3021 | ns |
|  | **F1 Unsupplemented** | **1** | Overall | | <0.0001 | **** |
|  |  |  | 0 (Control) | 0.05 | <0.0001 | **** |
|  |  |  | 0 (Control) | 0.5 | 0.3550 | ns |
|  |  |  | 0 (Control) | 5 | >0.9999 | ns |
|  |  |  | 0 (Control) | 50 | 0.0035 | ** |
|  |  |  | 0.05 | 0.5 | 0.0001 | *** |
|  |  |  | 0.05 | 5 | <0.0001 | **** |
|  |  |  | 0.05 | 50 | 0.0485 | * |
|  |  |  | 0.5 | 5 | 0.2919 | ns |
|  |  |  | 0.5 | 50 | 0.0513 | ns |
|  |  |  | 5 | 50 | 0.0018 | ** |
|  |  | **2** | Overall | | 0.0017 | ** |
|  |  |  | 0 (Control) | 0.05 | 0.2622 | ns |
|  |  |  | 0 (Control) | 0.5 | 0.0001 | *** |
|  |  |  | 0 (Control) | 5 | 0.0241 | * |
|  |  |  | 0 (Control) | 50 | 0.0170 | * |
|  |  |  | 0.05 | 0.5 | 0.0071 | ** |
|  |  |  | 0.05 | 5 | 0.3003 | ns |
|  |  |  | 0.05 | 50 | 0.2422 | ns |
|  |  |  | 0.5 | 5 | 0.0833 | ns |
|  |  |  | 0.5 | 50 | 0.1101 | ns |
|  |  |  | 5 | 50 | >0.9999 | ns |
| **Blood** | **F0** | **1** | Overall |  | 0.0159 | * |
|  |  |  | 0 (Control) | 0.05 | 0.1620 | ns |
|  |  |  | 0 (Control) | 0.1 | 0.6847 | ns |
|  |  |  | 0 (Control) | 0.5 | 0.0451 | * |
|  |  |  | 0 (Control) | 1 | 0.5744 | ns |
|  |  |  | 0.05 | 0.1 | 0.0599 | ns |
|  |  |  | 0.05 | 0.5 | 0.0007 | *** |
|  |  |  | 0.05 | 1 | 0.0433 | * |
|  |  |  | 0.1 | 0.5 | 0.1292 | ns |
|  |  |  | 0.1 | 1 | 0.9364 | ns |
|  |  |  | 0.5 | 1 | 0.1556 | ns |
|  |  | **2** | Overall |  | 0.0817 | ns |
|  | **F1 Supplemented** | **1** | Overall |  | 0.0006 | *** |
|  |  |  | 0 (Control) | 0.05 | 0.0142 | * |
|  |  |  | 0 (Control) | 0.1 | <0.0001 | **** |
|  |  |  | 0 (Control) | 0.5 | 0.5560 | ns |
|  |  |  | 0 (Control) | 1 | 0.0872 | ns |
|  |  |  | 0.05 | 0.1 | 0.1426 | ns |
|  |  |  | 0.05 | 0.5 | 0.0801 | ns |
|  |  |  | 0.05 | 1 | 0.4712 | ns |
|  |  |  | 0.1 | 0.5 | 0.0018 | ** |
|  |  |  | 0.1 | 1 | 0.0313 | * |
|  |  |  | 0.5 | 1 | 0.3345 | ns |
|  |  | **2** | Overall |  | 0.0490 | * |
|  |  |  | 0 (Control) | 0.05 | >0.9999 | ns |
|  |  |  | 0 (Control) | 0.1 | >0.9999 | ns |
|  |  |  | 0 (Control) | 0.5 | 0.0923 | ns |
|  |  |  | 0 (Control) | 1 | 0.0189 | * |
|  |  |  | 0.05 | 0.1 | >0.9999 | ns |
|  |  |  | 0.05 | 0.5 | 0.1199 | ns |
|  |  |  | 0.05 | 1 | 0.0248 | * |
|  |  |  | 0.1 | 0.5 | 0.1243 | ns |
|  |  |  | 0.1 | 1 | 0.0198 | * |
|  |  |  | 0.5 | 1 | 0.5543 | ns |
|  | **F1 Unsupplemented** | **1** | Overall |  | 0.0566 | ns |
|  |  | **2** | Overall |  | 0.8274 | ns |

**Supplementary Table 3.** Differences in mean clutch size between *A. stephensi* supplemented with Pan via water or blood. Data were analyzed by One-Way ANOVA (α = 0.05). * p < 0.05, ** p < 0.01, *** p < 0.001, **** p < 0.0001.

| **Pan Source** | **Generation** | **Gonotrophic Cycle** | **Treatment 1** | **Treatment 2** | **p-value** | **Significance** |
| --- | --- | --- | --- | --- | --- | --- |
| **Water** | **F0** | **1** | Overall |  | 0.2069 | ns |
|  |  | **2** | Overall |  | 0.0436 | ns |
|  |  |  | Control | 0.05 | 0.9998 | ns |
|  |  |  | Control | 0.1 | 0.9075 | ns |
|  |  |  | Control | 0.5 | 0.2215 | ns |
|  |  |  | Control | 1 | 0.9790 | ns |
|  |  |  | 0.05 | 0.1 | 0.9453 | ns |
|  |  |  | 0.05 | 0.5 | 0.1291 | ns |
|  |  |  | 0.05 | 1 | 0.9419 | ns |
|  |  |  | 0.1 | 0.5 | 0.0347 | * |
|  |  |  | 0.1 | 1 | 0.6212 | ns |
|  |  |  | 0.5 | 1 | 0.5558 | ns |
|  | **F1 Supplemented** | **1** | Overall | | 0.0050 | ** |
|  |  |  | Control | 0.05 | 0.4818 | ns |
|  |  |  | Control | 0.1 | 0.7643 | ns |
|  |  |  | Control | 0.5 | 0.9688 | ns |
|  |  |  | Control | 1 | 0.3062 | ns |
|  |  |  | 0.05 | 0.1 | 0.0355 | * |
|  |  |  | 0.05 | 0.5 | 0.1529 | ns |
|  |  |  | 0.05 | 1 | 0.0031 | ** |
|  |  |  | 0.1 | 0.5 | 0.9843 | ns |
|  |  |  | 0.1 | 1 | 0.9397 | ns |
|  |  |  | 0.5 | 1 | 0.7029 | ns |
|  |  | **2** | Overall | | 0.2230 | ns |
|  | **F1 Unsupplemented** | **1** | Overall |  | 0.0011 | ** |
|  |  |  | Control | 0.05 | 0.0026 | ** |
|  |  |  | Control | 0.1 | 0.9780 | ns |
|  |  |  | Control | 0.5 | 0.0604 | ns |
|  |  |  | Control | 1 | 0.2403 | ns |
|  |  |  | 0.05 | 0.1 | 0.0119 | * |
|  |  |  | 0.05 | 0.5 | 0.8594 | ns |
|  |  |  | 0.05 | 1 | 0.4127 | ns |
|  |  |  | 0.1 | 0.5 | 0.1856 | ns |
|  |  |  | 0.1 | 1 | 0.5375 | ns |
|  |  |  | 0.5 | 1 | 0.9552 | ns |
|  |  | **2** | Overall |  | 0.0002 | *** |
|  |  |  | Control | 0.05 | 0.0077 | ** |
|  |  |  | Control | 0.1 | 0.8724 | ns |
|  |  |  | Control | 0.5 | 0.9999 | ns |
|  |  |  | Control | 1 | 0.9714 | ns |
|  |  |  | 0.05 | 0.1 | <0.0001 | **** |
|  |  |  | 0.05 | 0.5 | 0.0077 | ** |
|  |  |  | 0.05 | 1 | 0.0282 | * |
|  |  |  | 0.1 | 0.5 | 0.7689 | ns |
|  |  |  | 0.1 | 1 | 0.4496 | ns |
|  |  |  | 0.5 | 1 | 0.9884 | ns |
| **Blood** | **F0** | **1** | Overall |  | 0.0009 | *** |
|  |  |  | Control | 0.05 | 0.9970 | ns |
|  |  |  | Control | 0.1 | 0.8390 | ns |
|  |  |  | Control | 0.5 | >0.9999 | ns |
|  |  |  | Control | 1 | 0.0026 | ** |
|  |  |  | 0.05 | 0.1 | 0.9490 | ns |
|  |  |  | 0.05 | 0.5 | 0.9972 | ns |
|  |  |  | 0.05 | 1 | 0.0062 | ** |
|  |  |  | 0.1 | 0.5 | 0.8511 | ns |
|  |  |  | 0.1 | 1 | 0.0950 | ns |
|  |  |  | 0.5 | 1 | 0.0039 | ** |
|  |  | **2** | Overall |  | 0.0081 | ** |
|  |  |  | Control | 0.05 | 0.1179 | ns |
|  |  |  | Control | 0.1 | 0.0359 | * |
|  |  |  | Control | 0.5 | 0.9169 | ns |
|  |  |  | Control | 1 | 0.9997 | ns |
|  |  |  | 0.05 | 0.1 | 0.9874 | ns |
|  |  |  | 0.05 | 0.5 | 0.5826 | ns |
|  |  |  | 0.05 | 1 | 0.1456 | ns |
|  |  |  | 0.1 | 0.5 | 0.3034 | ns |
|  |  |  | 0.1 | 1 | 0.0443 | * |
|  |  |  | 0.5 | 1 | 0.9577 | ns |
|  | **F1 Supplemented** | **1** | Overall |  | <0.0001 | **** |
|  |  |  | Control | 0.05 | 0.0005 | *** |
|  |  |  | Control | 0.1 | 0.6583 | ns |
|  |  |  | Control | 0.5 | 0.9643 | ns |
|  |  |  | Control | 1 | 0.4748 | ns |
|  |  |  | 0.05 | 0.1 | 0.1090 | ns |
|  |  |  | 0.05 | 0.5 | 0.0101 | * |
|  |  |  | 0.05 | 1 | <0.0001 | **** |
|  |  |  | 0.1 | 0.5 | 0.9573 | ns |
|  |  |  | 0.1 | 1 | 0.0399 | * |
|  |  |  | 0.5 | 1 | 0.1755 | ns |
|  |  | **2** | Overall |  | 0.0468 | * |
|  |  |  | Control | 0.05 | 0.9332 | ns |
|  |  |  | Control | 0.1 | 0.9999 | ns |
|  |  |  | Control | 0.5 | 0.7385 | ns |
|  |  |  | Control | 1 | 0.4387 | ns |
|  |  |  | 0.05 | 0.1 | 0.8763 | ns |
|  |  |  | 0.05 | 0.5 | 0.9921 | ns |
|  |  |  | 0.05 | 1 | 0.1011 | ns |
|  |  |  | 0.1 | 0.5 | 0.6397 | ns |
|  |  |  | 0.1 | 1 | 0.5347 | ns |
|  |  |  | 0.5 | 1 | 0.0355 | * |
|  | **F1 Unsupplemented** | **1** | Overall |  | 0.0066 | ** |
|  |  |  | Control | 0.05 | >0.9999 | ns |
|  |  |  | Control | 0.1 | 0.5075 | ns |
|  |  |  | Control | 0.5 | 0.2730 | ns |
|  |  |  | Control | 1 | 0.9348 | ns |
|  |  |  | 0.05 | 0.1 | 0.4026 | ns |
|  |  |  | 0.05 | 0.5 | 0.2614 | ns |
|  |  |  | 0.05 | 1 | 0.9451 | ns |
|  |  |  | 0.1 | 0.5 | 0.0024 | ** |
|  |  |  | 0.1 | 1 | 0.0891 | ns |
|  |  |  | 0.5 | 1 | 0.6788 | ns |
|  |  | **2** | Overall |  | 0.4946 | ns |

**Supplementary Table 4.** Mean clutch size ± standard error for each gonotrophic cycle (GC) following supplementation with Pan via water or blood.

| **Pan Source** | **Generation** | **Concentration (g/L)** | **GC 1** | **GC 2** |
| --- | --- | --- | --- | --- |
| **Water** | **F0** | 0 (Control) | 60.23 ± 1.770 | 56.83 ± 1.777 |
|  |  | 0.05 | 57.14 ± 1.711 | 57.22 ± 1.636 |
|  |  | 0.5 | 59.23 ± 1.878 | 59.10 ± 1.679 |
|  |  | 5 | 60.39 ± 1.729 | 51.75 ± 1.839 |
|  |  | 50 | 55.31 ± 1.815 | 55.41 ± 1.668 |
|  | **F1 Supplemented** | 0 (Control) | 58.49 ± 1.527 | 60.56 ± 1.778 |
|  |  | 0.05 | 61.60 ± 1.336 | 63.07 ± 1.493 |
|  |  | 0.5 | 56.23 ± 1.375 | 63.62 ± 1.533 |
|  |  | 5 | 57.25 ± 1.347 | 60.97 ± 1.797 |
|  |  | 50 | 54.77 ± 1.172 | 58.81 ± 1.738 |
|  | **F1 Unsupplemented** | 0 (Control) | 50.84 ± 1.537 | 61.65 ± 1.813 |
|  |  | 0.05 | 58.48 ± 1.379 | 70.22 ± 2.120 |
|  |  | 0.5 | 52.08 ± 1.416 | 59.21 ± 1.703 |
|  |  | 5 | 56.51 ± 1.373 | 62.04 ± 1.411 |
|  |  | 50 | 55.12 ± 1.437 | 63.18 ± 1.595 |
| **Blood** | **F0** | 0 (Control) | 63.15 ± 1.597 | 58.64 ± 1.870 |
|  |  | 0.05 | 62.37 ± 1.810 | 52.14 ± 2.001 |
|  |  | 0.1 | 60.23 ± 1.678 | 50.80 ± 1.813 |
|  |  | 0.5 | 63.17 ± 1.863 | 56.23 ± 1.910 |
|  |  | 1 | 54.55 ± 1.418 | 58.14 ± 1.953 |
|  | **F1 Supplemented** | 0 (Control) | 56.02 ± 1.489 | 55.12 ± 1.736 |
|  |  | 0.05 | 64.95 ± 1.694 | 57.25 ± 2.084 |
|  |  | 0.1 | 59.18 ± 1.459 | 54.70 ± 1.751 |
|  |  | 0.5 | 57.50 ± 1.688 | 58.46 ± 2.048 |
|  |  | 1 | 52.29 ± 1.801 | 50.55 ± 1.964 |
|  | **F1 Unsupplemented** | 0 (Control) | 58.74 ± 1.945 | 60.70 ± 2.142 |
|  |  | 0.05 | 58.94 ± 1.710 | 56.02 ± 1.892 |
|  |  | 0.1 | 54.53 ± 1.746 | 56.25 ± 1.819 |
|  |  | 0.5 | 64.13 ± 2.023 | 55.72 ± 2.271 |
|  |  | 1 | 60.77 ± 1.689 | 57.30 ± 2.608 |

**Supplementary Table 5.** Differences in offspring sex ratio between *A. stephensi* supplemented with Pan via water or blood. Data were analyzed by Chi-Square test (α = 0.05). * p < 0.05, ** p < 0.01, *** p < 0.001, **** p < 0.0001.

| **Pan Source** | **Generation** | **Gonotrophic Cycle** | **Treatment 1 (g/L)** | **Treatment 2 (g/L)** | **p-value** | **Significance** |
| --- | --- | --- | --- | --- | --- | --- |
| **Water** | **F0** | **1** | Overall | | 0.0026 | ** |
|  |  |  | 0 (Control) | 0.05 | 0.0005 | *** |
|  |  |  | 0 (Control) | 0.5 | 0.6125 | ns |
|  |  |  | 0 (Control) | 5 | 0.6833 | ns |
|  |  |  | 0 (Control) | 50 | 0.9719 | ns |
|  |  |  | 0.05 | 0.5 | 0.0074 | ** |
|  |  |  | 0.05 | 5 | 0.0020 | ** |
|  |  |  | 0.05 | 50 | 0.0009 | *** |
|  |  |  | 0.5 | 5 | 0.8995 | ns |
|  |  |  | 0.5 | 50 | 0.6039 | ns |
|  |  |  | 5 | 50 | 0.6735 | ns |
|  |  | **2** | Overall | | <0.0001 | **** |
|  |  |  | 0 (Control) | 0.05 | 0.0556 | ns |
|  |  |  | 0 (Control) | 0.5 | 0.8965 | ns |
|  |  |  | 0 (Control) | 5 | <0.0001 | **** |
|  |  |  | 0 (Control) | 50 | 0.4897 | ns |
|  |  |  | 0.05 | 0.5 | 0.0487 | * |
|  |  |  | 0.05 | 5 | 0.0001 | *** |
|  |  |  | 0.05 | 50 | 0.1955 | ns |
|  |  |  | 0.5 | 5 | <0.0001 | **** |
|  |  |  | 0.5 | 50 | 0.4270 | ns |
|  |  |  | 5 | 50 | <0.0001 | **** |
|  | **F1 Supplemented** | **1** | Overall | | <0.0001 | **** |
|  |  |  | Control | 0.05 | 0.0914 | ns |
|  |  |  | Control | 0.5 | 0.9510 | ns |
|  |  |  | Control | 5 | 0.0019 | ** |
|  |  |  | Control | 50 | 0.6195 | ns |
|  |  |  | 0.05 | 0.5 | 0.1002 | ns |
|  |  |  | 0.05 | 5 | <0.0001 | **** |
|  |  |  | 0.05 | 50 | 0.2535 | ns |
|  |  |  | 0.5 | 5 | 0.0013 | ** |
|  |  |  | 0.5 | 50 | 0.6533 | ns |
|  |  |  | 5 | 50 | 0.0003 | *** |
|  |  | **2** | Overall | | 0.0012 | ** |
|  |  |  | Control | 0.05 | 0.0141 | * |
|  |  |  | Control | 0.5 | 0.2924 | ns |
|  |  |  | Control | 5 | 0.0386 | * |
|  |  |  | Control | 50 | 0.7404 | ns |
|  |  |  | 0.05 | 0.5 | 0.0003 | *** |
|  |  |  | 0.05 | 5 | 0.7562 | ns |
|  |  |  | 0.05 | 50 | 0.0430 | * |
|  |  |  | 0.5 | 5 | 0.0015 | ** |
|  |  |  | 0.5 | 50 | 0.1758 | ns |
|  |  |  | 5 | 50 | 0.1008 | ns |
|  | **F1 Unsupplemented** | **1** | Overall | | 0.3390 | ns |
|  |  | **2** | Overall | | <0.0001 | **** |
|  |  |  | Control | 0.05 | 0.0008 | *** |
|  |  |  | Control | 0.5 | 0.1039 | ns |
|  |  |  | Control | 5 | 0.4191 | ns |
|  |  |  | Control | 50 | 0.0162 | * |
|  |  |  | 0.05 | 0.5 | <0.0001 | **** |
|  |  |  | 0.05 | 5 | 0.0127 | * |
|  |  |  | 0.05 | 50 | 0.2697 | ns |
|  |  |  | 0.5 | 5 | 0.0165 | * |
|  |  |  | 0.5 | 50 | <0.0001 | **** |
|  |  |  | 5 | 50 | 0.1257 | ns |
| **Blood** | **F0** | **1** | Overall | <0.0001 | **** |  |
|  |  |  | 0 (Control) | 0.05 | 0.4304 | ns |
|  |  |  | 0 (Control) | 0.1 | 0.0191 | * |
|  |  |  | 0 (Control) | 0.5 | 0.0701 | ns |
|  |  |  | 0 (Control) | 1 | 0.0315 | * |
|  |  |  | 0.05 | 0.1 | 0.0019 | ** |
|  |  |  | 0.05 | 0.5 | 0.2814 | ns |
|  |  |  | 0.05 | 1 | 0.0036 | ** |
|  |  |  | 0.1 | 0.5 | <0.0001 | **** |
|  |  |  | 0.1 | 1 | 0.8232 | ns |
|  |  |  | 0.5 | 1 | 0.0002 | *** |
|  |  | **2** | Overall | <0.0001 | **** |  |
|  |  |  | 0 (Control) | 0.05 | 0.5018 | ns |
|  |  |  | 0 (Control) | 0.1 | 0.0994 | ns |
|  |  |  | 0 (Control) | 0.5 | 0.0020 | ** |
|  |  |  | 0 (Control) | 1 | 0.0202 | * |
|  |  |  | 0.05 | 0.1 | 0.0241 | * |
|  |  |  | 0.05 | 0.5 | 0.0154 | * |
|  |  |  | 0.05 | 1 | 0.0032 | ** |
|  |  |  | 0.1 | 0.5 | <0.0001 | **** |
|  |  |  | 0.1 | 1 | 0.5931 | ns |
|  |  |  | 0.5 | 1 | <0.0001 | **** |
|  | **F1 Supplemented** | **1** | Overall | 0.0018 | ** |  |
|  |  |  | 0 (Control) | 0.05 | 0.0733 | ns |
|  |  |  | 0 (Control) | 0.1 | 0.1620 | ns |
|  |  |  | 0 (Control) | 0.5 | 0.0451 | * |
|  |  |  | 0 (Control) | 1 | 0.5945 | ns |
|  |  |  | 0.05 | 0.1 | 0.7295 | ns |
|  |  |  | 0.05 | 0.5 | 0.0002 | *** |
|  |  |  | 0.05 | 1 | 0.2435 | ns |
|  |  |  | 0.1 | 0.5 | 0.0009 | *** |
|  |  |  | 0.1 | 1 | 0.4224 | ns |
|  |  |  | 0.5 | 1 | 0.0147 | * |
|  |  | **2** | Overall | 0.0002 | *** |  |
|  |  |  | 0 (Control) | 0.05 | 0.1134 | ns |
|  |  |  | 0 (Control) | 0.1 | 0.6124 | ns |
|  |  |  | 0 (Control) | 0.5 | 0.0058 | * |
|  |  |  | 0 (Control) | 1 | 0.1775 | ns |
|  |  |  | 0.05 | 0.1 | 0.0344 | * |
|  |  |  | 0.05 | 0.5 | <0.0001 | **** |
|  |  |  | 0.05 | 1 | 0.8649 | ns |
|  |  |  | 0.1 | 0.5 | 0.0189 | * |
|  |  |  | 0.1 | 1 | 0.0611 | ns |
|  |  |  | 0.5 | 1 | <0.0001 | **** |
|  | **F1 Unsupplemented** | **1** | Overall |  | 0.0050 | ** |
|  |  |  | 0 (Control) | 0.05 | 0.8394 | ns |
|  |  |  | 0 (Control) | 0.1 | 0.9251 | ns |
|  |  |  | 0 (Control) | 0.5 | 0.3961 | ns |
|  |  |  | 0 (Control) | 1 | 0.0018 | ** |
|  |  |  | 0.05 | 0.1 | 0.7611 | ns |
|  |  |  | 0.05 | 0.5 | 0.5106 | ns |
|  |  |  | 0.05 | 1 | 0.0036 | ** |
|  |  |  | 0.1 | 0.5 | 0.3495 | ns |
|  |  |  | 0.1 | 1 | 0.0020 | ** |
|  |  |  | 0.5 | 1 | 0.0501 | ns |
|  |  | **2** | Overall | 0.0701 | ns |  |
